# Swallowing disorders in cerebral palsy: a systematic review of oropharyngeal Dysphagia, nutritional impact, and health risks

**DOI:** 10.1186/s13052-025-01903-1

**Published:** 2025-02-22

**Authors:** Andrea Calderone, David Militi, Davide Cardile, Francesco Corallo, Rocco Salvatore Calabrò, Angela Militi

**Affiliations:** 1 IRCCS Centro Neurolesi Bonino-Pulejo, Via Palermo, C.da Casazza, S.S. 113, Messina, 98124 Italy; 2 Department of Biomedical and Dental Sciences and Morphological and Functional Imaging, University of Messina, Messina, 98125 Italy

**Keywords:** Cerebral Palsy, Oropharyngeal Dysphagia, Nutritional Status, Swallowing Disorders, Health Complications, Neurorehabilitation

## Abstract

Cerebral palsy (CP) is a permanent disorder affecting movement and posture due to nonprogressive brain issues, often leading to various sensory, cognitive, and musculoskeletal challenges. Among these complications, oropharyngeal dysphagia (OPD) is prevalent, impacting up to 85% of children with CP and resulting in significant nutritional deficits. This systematic review aims to explore the prevalence and types of OPD in CP patients, its effects on nutritional status, and its associated health complications, emphasizing the need for thorough assessment and intervention to mitigate risks. The review adheres to PRISMA guidelines, searching five major databases (PubMed, Web of Science, Embase, Cochrane Library, and Scopus) without time range restrictions to capture studies addressing swallowing disorders and their impact on nutritional status in CP. This review has been registered on Open OSF (n) 3KUQX. Individuals with CP often experience swallowing impairments, including delayed pharyngeal transit and aspiration. Research indicates that 81.5% of kids with CP suffer from dysphagia, commonly associated with reduced motor skills and general health. Moreover, as a result of these swallowing difficulties, nutritional complications may occur, with elevated levels of gastroesophageal symptoms causing malnutrition and growth delays, which require thorough evaluations and personalized interventions for successful treatment. Tools like the Videofluoroscopic Swallowing Study were identified as primary methods for evaluation, but assessment remains limited by methodological inconsistencies. This systematic review underscores the significant health impacts of OPD in children with CP, which affects nutrition and overall well-being. Future research should address the need for standardized evaluation methods and effective interventions to balance nutritional needs with practical mealtime strategies.

## Introduction

Cerebral palsy (CP) is a nonprogressive neurological disorder affecting movement and posture, often accompanied by oropharyngeal dysphagia (OPD), a significant contributor to malnutrition and health complications [1, 2]. Data from developed countries indicate that the occurrence of CP is 2–3 per 1000 live births, with a much higher rate of 40–100 per 1000 in premature babies born before 28 weeks of gestation [3–8]. Motor impairments in CP affect oral and pharyngeal coordination, leading to aspiration and pulmonary complications [9–12]. In fact, OPD affects up to 85% of children with CP and is connected to reduced food consumption [13–17]. The operational definition of OPD is impairment in the swallowing function, as described in the International Classification of Functional Health Disorders (ICF) as "related to the ingestion and handling of solids or liquids from the mouth into the body" [18, 19]. The ICF defines eating and drinking as a combination of the upper extremity function, social and cultural elements, and the swallowing function. OPD disrupts all phases of swallowing: oral, pharyngeal, and esophageal. Primitive reflexes like sucking and chewing remain, impairing bolus formation and safe swallowing [20, 21]. These difficulties are exacerbated by inadequate nourishment and hydration, resulting in extended, stressful mealtimes [22–24]. The severity of dysphagia correlates with motor and cognitive impairments. Therefore, effective treatment necessitates a multidisciplinary approach that considers gastrointestinal, respiratory, nutritional, and motor abilities, as well as behavioral and familiar factors [25–30]. A comprehensive assessment of both swallowing and nutrition is essential for optimal rehabilitation [31]. Table 1 provides an overview of the psychometric and diagnostic tools (including clinical and instrumental assessments of dysphagia) used to assess dietary and swallowing issues [32–67]. Given these challenges, understanding the swallowing process and its impact on nutrient intake is crucial. Swallowing is a complicated process involving synchronized muscle and nerve action in the oral cavity, throat, and esophagus to safely deliver food and liquids to the stomach [12, 68–70]. Children with CP commonly experience trouble eating due to physical and functional implications. This reduces energy, muscular mass, and immune function, raising the risk of infections and other repercussions [71–73]. Furthermore, swallowing dysfunctions can result in symptoms such as pharyngeal itching, coughing, hypersensitivity, choking, and vomiting, which can bring problems such as pneumonia, weight loss, cognitive impairment, and higher death rates [74–76]. Patients with CP may also have gastroesophageal reflux disease (GERD), which causes symptoms such as heartburn and regurgitation [76–81]. Despite OPD being a severe complication in CP, there is a notable lack of standardized assessment tools and integrated intervention strategies addressing both motor and nutritional aspects. Current diagnostic practices vary widely, often relying on fragmented methodologies that fail to capture the multidimensional nature of OPD, encompassing motor, sensory, and nutritional aspects. Additionally, integrated intervention strategies that address both swallowing dysfunction and its nutritional consequences remain underexplored. The scarcity of evidence-based guidelines for multidisciplinary management further underscores the need for a systematic synthesis of available knowledge. This systematic review is timely, addressing critical gaps by consolidating evidence on OPD's prevalence, clinical burden, and the lack of standardized, multidisciplinary approaches for its management. It aims to provide a clearer understanding of the disorder’s impact on nutrition, growth, and related health outcomes. Furthermore, it seeks to identify existing limitations in diagnostic practices and propose directions for future research, fostering the development of standardized approaches and integrated care models. Given the profound health implications of untreated OPD, a thorough synthesis of available data is essential to guide clinical decision-making and policy formulation.

**Table 1:** shows the psychometric tool and diagnostic instruments for dietary and swallowing issues

**Tool Name**	**Tool Type (Clinical or Instrumental Assessment)**	**Description**
Oropharyngeal Dysphagia Scale (ODS)	Clinical Assessment	ODS is a psychometric measure developed primarily to assess the severity of oropharyngeal dysphagia in people who have difficulty swallowing. It has ten items, each addressing a distinct component of swallowing function, such as bolus management, aspiration risk, pharyngeal residue, and swallowing start. Each issue is rated on a scale of 0 to 3, with higher scores indicating more dysfunction [32]
Eating and Drinking Ability Classification System (EDACS)	Clinical Assessment	The EDACS evaluates the functional capacity to eat and drink in people with CP. It has five levels, with Level I signifying complete independence and Level V requiring full help. This measure assesses overall swallowing safety and efficiency, taking into account motor control, posture, and food texture adjustments to facilitate safe and effective intake [33]
Feeding Swallowing Impact Survey (FSIS)	Clinical Assessment	The FSIS is a 33-item scale that assesses the impact of feeding and swallowing issues on the quality of life for children and caregivers. It addresses issues such as physical pain, emotional effects, and social elements of eating. Responses are assessed on a 5-point Likert scale, making it an effective tool for examining the larger implications of feeding concerns [34]
Clinical Dysphagia Scale (CDS)	Clinical Assessment	The CDS is an 8-item scale that assesses the severity of dysphagia using clinical observations during swallowing. It evaluates characteristics including cough, voice alteration, and oral and pharyngeal residue. Each question is rated on a scale of 0 to 4, allowing doctors to assess the severity of dysphagia and guide treatment planning [35]
Penetration-Aspiration Scale (PAS)	Instrumental Assessment	The PAS is an 8-point scale that assesses the severity of debris entering the airway when swallowing. The scores range from 1 (no penetration or aspiration) to 8 (silent aspiration) and serve as a measure of swallowing safety. This scale is commonly used in videofluoroscopic swallowing investigations to evaluate the risk of aspiration-related problems [36]
Functional Oral Intake Scale (FOIS)	Clinical Assessment	The FOIS determines the level of oral intake in people with swallowing difficulties. It has seven levels, ranging from no oral consumption (Level 1) to entire oral intake without limits (Level 7). The FOIS is very beneficial for tracking patient improvement and assessing the need for dietary changes [37–39]
Videofluoroscopic Swallowing Studies (VFSS)	Instrumental Assessment	VFSS is a tool for visualizing the swallowing process using X-rays. It assesses all stages of swallowing and detects anomalies such as aspiration or residue in various food and beverage consistencies. VFSS is crucial for identifying dysphagia and creating personalized treatment programs [40–43]
Videofluoroscopic Dysphagia Scale (VDS)	Instrumental Assessment	The VDS is a 14-item scale used to measure the severity of dysphagia using videofluoroscopic images. Each question assesses a specific element, such as pharyngeal transit time and aspiration, with scores reflecting overall swallowing performance. This scale helps to track progress and guide dysphagia therapy [44, 45]
Dysphagia Management Staging Scale (DMSS)	Clinical Assessment	The DMSS divides dysphagia management requirements into phases to help guide therapeutic actions. It assesses elements such as feeding assistance and nutrition modification, ranging from independent (Stage 1) to entirely reliant on specialist intervention (Stage 5). This scale helps doctors design suitable dysphagia management techniques [46]
Swallow Function Scales (SFS)	Clinical Assessment	The SFS is a series of measures used to evaluate functional elements of swallowing, such as bolus control and airway protection. Items are rated on a scale of 0 to 4, with higher scores indicating more disability. The SFS offers a formal framework for evaluating and monitoring swallowing function throughout time [47]
Food Intake Level Scale (FILS)	Clinical Assessment	The FILS is a 10-point scale for measuring oral meal intake in people with swallowing difficulties. Levels range from no oral intake (Level 1) to complete consumption of a typical diet (Level 10). The FILS is useful for assessing food limitations and tracking changes in swallowing function [48]
Fibreoptic Endoscopic Evaluation of Swallowing (FEES)	Instrumental Assessment	The FEES utilizes a flexible fiberoptic endoscope to directly observe the swallowing process, and is commonly used for diagnosis. It offers thorough information on the anatomy and physiology of the pharyngeal swallowing phase, aiding clinicians in detecting problems like aspiration, penetration, and residue. This approach is especially successful in assessing individuals with neurological conditions, structural irregularities, or unclear swallowing issues. FEES is a low-impact procedure, radiation-free, and enables multiple real-time evaluations [49]
Fiberoptic Endoscopic Evaluation of Swallowing with Sensory Testing (FEESST)	Instrumental Assessment	It is a diagnostic procedure used to assess swallowing function and laryngeal sensory function. A thin, flexible endoscope is passed through the nose to visualize the pharynx and larynx during swallowing. In addition to observing the swallowing mechanism, FEESST involves delivering air pulses to the aryepiglottic folds to test the laryngeal adductor reflex, providing information about sensory function in the larynx. This helps identify sensory deficits that may contribute to swallowing difficulties [49]
Pediatric Dysphagia Risk Screening Instrument (PDRSI)	Clinical Assessment	The PDRSI is a handy screening tool created to identify initial signs of dysphagia in children's groups. It evaluates various factors such as eating habits, physical symptoms (such as coughing, choking), and past illnesses to pinpoint children who could be in danger. Healthcare professionals can easily use the device in clinical or educational environments due to its user-friendly interface. The PDRSI enables timely referrals for more thorough evaluations or interventions by detecting issues early [50]
Pediatric version of the Eating Assessment Tool-10 (PEDI-EAT-10)	Clinical Assessment	The PEDI-EAT-10 questionnaire is a validated tool designed for children, based on the adult Eating Assessment Tool. Caregivers complete it to evaluate how often and how severe children have swallowing and feeding issues. The tool consists of ten items that address problems like rejecting food, trouble swallowing, and extended feeding durations. It’s simple structure makes it a dependable tool for tracking symptoms longitudinally and informing medical judgments. The PEDI-EAT-10 is especially beneficial for recognizing kids with functional or neurological feeding issues [51]
Behavioral Assessment Scale of Oral Functions in Feeding (BASOFF)	Clinical Assessment	The BASOFF is a comprehensive evaluation instrument made to assess oral motor abilities and feeding habits in children facing feeding problems. It offers a organized system for evaluating important elements of oral functions such as chewing, swallowing, and coordinating oral movements. The tool is especially useful for detecting developmental delays or neurological issues that impact feeding. Its thorough scoring system enables clinicians to identify particular areas of dysfunction and monitor improvement as time goes on [52]
Signaleringslijst Verslikke (SV)	Clinical Assessment	The SV is a screening tool from the Netherlands that is made to identify people who may be at risk of aspiration while swallowing. It assesses visible signs like coughing, choking, and voice alterations post-swallowing, along with clinical indications of complications related to aspiration. The tool is especially beneficial for individuals with neurological conditions like CP, stroke or Parkinson’s disease, as well as in older patients. By pinpointing individuals at high risk, the SV enables prompt referrals for advanced diagnostic tests like FEES or videofluoroscopy. It also assists in creating customized management plans to avoid aspiration pneumonia [53]
Choking Risk Assessment (CRA) [54]	Clinical Assessment	The CRA is a specific instrument employed to assess the likelihood of choking while eating, specifically in people who have issues with swallowing. Factors such as food texture, swallowing mechanics, and the feeding environment are considered. The CRA plays a crucial role in recognizing individuals with a high risk, like those with neurological disorders, developmental delays, or structural abnormalities in the upper airway. The tool helps to identify possible choking risks, enabling the creation of safety measures for meals, like changing food textures or using particular feeding methods. Using it can greatly decrease the chances of dangerous choking situations, leading to improved safety for patients [54]
Nutrition and Swallow Checklist (NSC) [55]	Clinical Assessment	The NSC is a thorough assessment tool created to evaluate the nutritional status and swallowing function of people of all ages. It assesses various factors such as involuntary weight loss, indicators of difficulty swallowing, and food consumption. The checklist is easy to use, making it perfect for both clinical and home settings. The NSC helps by detecting people who are vulnerable to malnutrition or dehydration, allowing for early actions like adjusting diets or receiving swallowing treatments [55]
Screening Tool of Feeding Problems Scale (STEP) [56]	Clinical Assessment	The STEP is a reliable tool created to pinpoint feeding challenges in children, especially those with developmental disabilities or medical issues. It evaluates feeding habits, ability to use mouth muscles, and parent worries with a thorough survey. The STEP offers helpful information on the root causes of feeding issues, like sensory aversions or motor difficulties, which helps clinicians create specific interventions [56]
Dysphagia Disorders Survey (DDS)	Clinical Assessment	It evaluates various stages of swallowing, from the oral phase (tongue and lip movements) to the pharyngeal phase (bolus passage through the pharynx) and finally the esophageal phase (bolus passage through the esophagus). The DDS can involve direct observation, reflex testing, and the administration of foods and liquids of different consistencies to observe the patient's response. It is a valuable tool for pinpointing the location and severity of the swallowing disorder. It's important to note that while used with adults, modified versions may exist or components may be adapted for pediatric use [57]
Parent-reported Feeding and Swallowing Questionnaire (FSQ)	Clinical Assessment	This questionnaire relies on the observations of parents or caregivers, who are key figures in a child's feeding. Questions may cover the frequency of coughing or choking during meals, difficulty chewing or swallowing foods of certain textures, meal duration, the presence of regurgitation or vomiting, and the child's attitude towards food. The FSQ provides valuable insights into the child's eating behavior in their natural environment [58]
Dysphagia Risk Assessment Protocol (PARD)	Clinical Assessment	The PARD is not a diagnostic test but a screening tool to identify individuals at risk of developing dysphagia. This protocol considers factors such as age, the presence of neurological diseases (CP, stroke, Parkinson's, etc.), head or neck surgeries, prolonged intubation, and other medical conditions that may predispose someone to dysphagia. Early identification of risk allows for timely preventive or diagnostic interventions [59]
Volume Viscosity Swallowing Test (V-VST)	Clinical Assessment	This test focuses on assessing the patient's ability to manage different volumes and consistencies of food and liquids. The patient is offered small amounts of liquids (e.g., 5 ml) and then larger amounts (e.g., 10 ml, 20 ml) of varying viscosities (water, nectar, honey, pudding). The clinician observes for coughing, wet voice, difficulty managing the bolus, and other signs of swallowing difficulty. The V-VST helps determine the safest diet for the patient [60]
Functional Dysphagia Scale (FDS)	Instrumental Assessment	This scale evaluates the impact of dysphagia on the patient's daily life. It considers the ability to eat independently, the need for assistance during meals, the presence of complications such as aspiration pneumonia, and the patient's quality of life. The FDS is useful for monitoring patient progress during rehabilitation [61]
Oral Hygiene Index Simplified (OHIS)	Clinical Assessment	While not specifically a dysphagia test, the OHIS is important in the context of dysphagia management. Poor oral hygiene increases the risk of lung infections, particularly aspiration pneumonia, which is a serious complication of dysphagia. The OHIS assesses the amount of plaque and tartar present on the teeth, providing an indication of the patient's oral hygiene level [62]
Nordic Orofacial Test-Screening (NOT-S)	Clinical Assessment	This is a quick and easy-to-administer screening tool designed to identify potential orofacial dysfunction in individuals of all ages, though it's particularly useful in pediatric populations. It assesses various aspects of oromotor function, including lip closure, tongue mobility and strength, jaw movements, and facial sensitivity. The NOT-S provides a standardized method for observing and scoring these functions, allowing clinicians to quickly identify individuals who may require further, more in-depth assessment of their feeding and swallowing abilities [63]
Multichannel intraluminal impedance (MII)	Instrumental Assessment	is a technique used to evaluate esophageal function by measuring changes in electrical impedance within the esophagus. These changes reflect the passage of a bolus (food or liquid) through the esophagus. Unlike pH monitoring, which only detects acid reflux, MII can detect all types of refluxes, including non-acid reflux (e.g., liquid or gas). This is particularly important for individuals with dysphagia who may experience reflux of non-acidic contents that can irritate the esophagus and contribute to swallowing problems. MII is often used in conjunction with esophageal manometry (a test that measures esophageal muscle contractions) and pH monitoring to provide a comprehensive assessment of esophageal function [64]
Viking Speech Scale (VSS)	Clinical Assessment	It is a valuable tool for assessing speech production, particularly in individuals with dysarthria (motor speech disorders). Dysarthria can often co-occur with dysphagia because both conditions involve impairments in the muscles used for speech and swallowing. The VSS evaluates various aspects of speech, including articulation (the clarity of speech sounds), phonation (voice production), resonance (the quality of the voice), and prosody (the rhythm and intonation of speech). By assessing these speech components, clinicians can gain insights into the individual's oral motor control and coordination, which can be relevant to swallowing function [65]
Ultrasound Imaging of Swallowing (SWUS)	Instrumental Assessment	SWUS is a non-invasive and radiation-free imaging technique that uses high-frequency sound waves to create real-time images of the oral phase of swallowing. It allows clinicians to visualize tongue movements, hyoid bone excursion (the movement of a bone in the neck that is important for swallowing), and bolus formation and transport. SWUS is particularly useful for assessing oral motor dysfunction and can be used to provide biofeedback during therapy. However, it has limitations in visualizing the pharyngeal and esophageal phases of swallowing, as these structures are obscured by bone and air [66]
Cervical Auscultation (CA)	Clinical Assessment	It is a simple and inexpensive method of assessing swallowing by listening to the sounds produced during swallowing using a stethoscope placed on the neck. Clinicians listen for specific sounds, such as the timing of the swallow, the presence of wet or gurgly sounds (which may indicate aspiration), and the presence of stridor (a high-pitched whistling sound that may indicate airway obstruction). While CA can provide some clues about swallowing function, it is a subjective method and has limitations in its accuracy and reliability. It should not be used as a standalone diagnostic tool but rather as a component of a comprehensive swallowing assessment [67]

Legend: *ODS* Oropharyngeal Dysphagia Scale, *EDACS* Eating and Drinking Ability Classification System, *CP* Cerebral Palsy, *FSIS* Feeding Swallowing Impact Scale, *CDS* Clinical Dysphagia Scale, *PAS* Penetration-Aspiration Scale, *FOIS* Functional Oral Intake Scale, *VFSS* Videofluoroscopic Swallowing Studies, *VDS* Videofluoroscopic Dysphagia Scale, *DMSS* Dysphagia Management Staging Scale, *SFS* Swallow Function Scales, *FILS* Food Intake Level Scale, *FEES* Fibreoptic Endoscopic Evaluation of Swallowing, *FEESST* Fiberoptic Endoscopic Evaluation of Swallowing with Sensory Testing, *PDRSI* Pediatric Dysphagia Risk Screening Instrument, *PEDI-EAT-10* Pediatric version of the Eating Assessment Tool-10, *BASOFF* Behavioral Assessment Scale of Oral Functions in Feeding, *SV* Signaleringslijst Verslikke, *CRA* Choking Risk Assessment, *NSC* Nutrition and Swallow Checklist, *STEP* Screening Tool of Feeding Problems Scale, *DDS* Dysphagia Disorders Survey, *FSQ* Parent-reported Feeding and Swallowing Questionnaire, *PARD* Dysphagia Risk Assessment Protocol, *V-VST* Volume Viscosity Swallowing Test, *FDS* Functional Dysphagia Scale, *OHIS* Oral Hygiene Index Simplified, *NOT-S* Nordic Orofacial Test-Screening, *MII* Multichannel intraluminal impedance, *pH* Potential of Hydrogen, *VSS* Viking Speech Scale, *SWUS* Ultrasound Imaging of Swallowing, *CA* Cervical Auscultation

## Materials and methods

### Search strategy

**Fig. 1:**
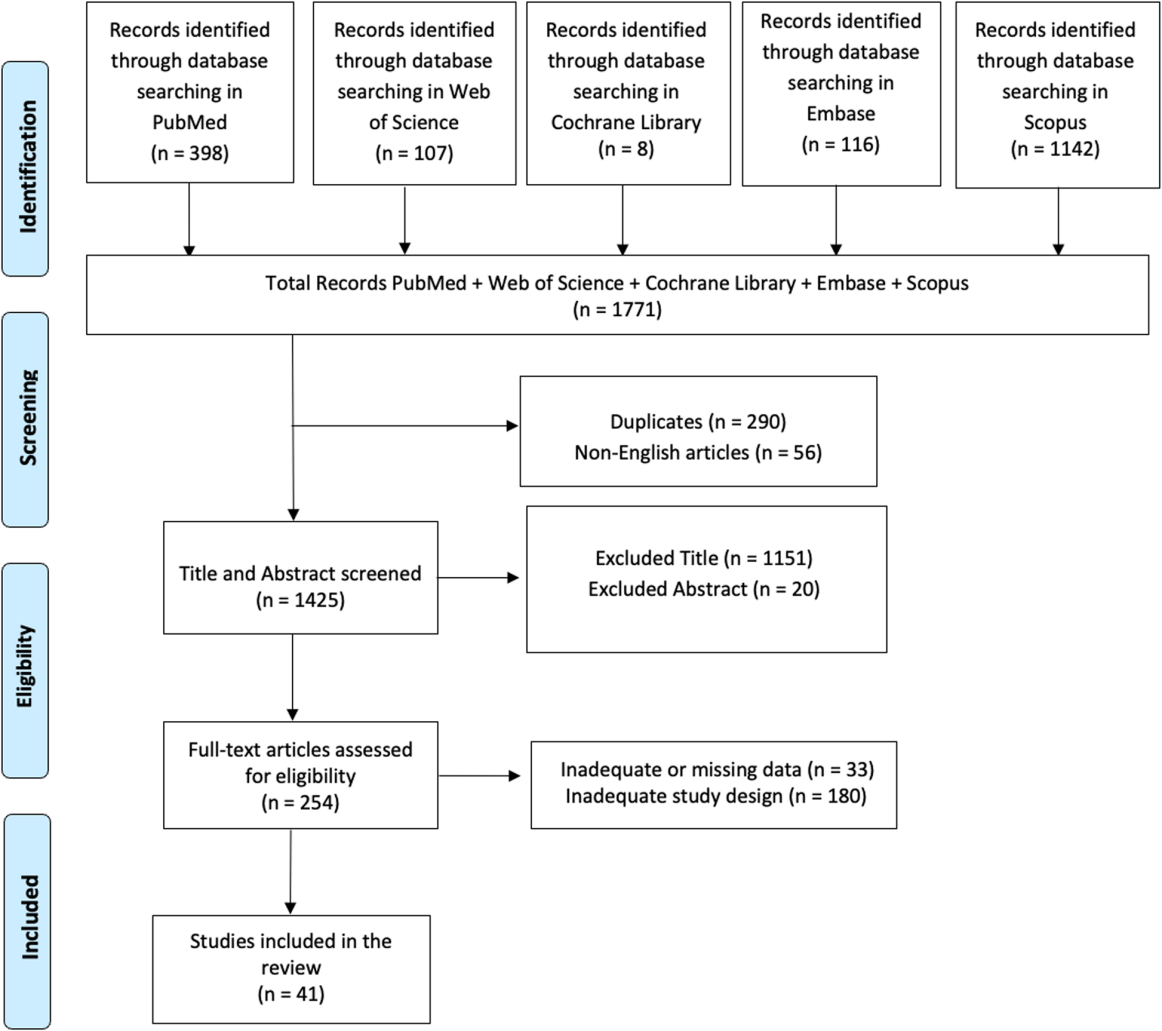
PRISMA 2020 flow diagram of evaluated studies

A literature search was conducted using PubMed, Web of Science, Cochrane Library, Embase, and Scopus databases, employing the keywords: (Oropharyngeal Dysphagia) AND/OR (Cerebral Palsy) AND/OR (Nutritional status). The investigation was carried out with no limitations of time, enabling a thorough examination of the changing knowledge about OPD in children with CP. Searches were conducted independently by two reviewers (AC, DM) using Boolean operators and controlled vocabulary (e.g., MeSH terms), to enhance transparency and accuracy in identifying relevant studies. The PRISMA (Preferred Reporting Items for Systematic Reviews and Meta-Analyses) flow diagram was utilized to outline the process (identification, screening, eligibility, and inclusion) for selecting relevant studies addressing the prevalence, types, and assessment of OPD and swallowing disorders in patients with CP as well as its impact on nutritional status and related health complications, as illustrated in Fig. 1 [82].

The Prisma Check list was also used to guarantee the scientific rigor of the paper (as shown in the supplementary materials). All articles were screened based on titles, abstracts, and full texts by the two researchers (AC, DM), who independently performed data extraction, article collection, and cross-validation to reduce the risk of bias (e.g., missing result bias, publication bias; time lag bias; language bias). These researchers read full-text articles deemed eligible for the study, and in case of disagreement on inclusion and exclusion criteria, the final decision was made by a third researcher (RSC). Moreover, the agreement between the two reviewers (AC and DM) was assessed using the kappa statistics. The kappa score, with an accepted threshold for substantial agreement set at > 0.61, was interpreted to reflect excellent concordance between the reviewers. This criterion ensures a robust evaluation of the inter-rater reliability, emphasizing the achievement of a substantial level of agreement in the data extraction process. The list of articles was then refined for relevance, reviewed, and summarized, with key topics identified from the summary based on the inclusion/exclusion criteria. This review was registered with a DOI (https://doi.org/10.17605/OSF.IO/8HJK4) on the Open Science Framework (OSF).

### PICO Evaluation

We applied the PICO model (Population, Intervention, Comparison, Outcome) to create our search terms.

In this review, the population focuses on individuals with CP who show symptoms of OPD and other swallowing problems. The intervention concentrates on different ways to evaluate OPD and associated swallowing issues, such as diagnostic assessments and screening instruments designed to identify swallowing difficulties in the group, as well as rehabilitation techniques. Due to the observational nature of the studies reviewed, we did not incorporate a particular comparison group. Nonetheless, research papers were chosen by looking at how swallowing issues affect the nutritional status and overall health of children and adults with CP in comparison to typical standards for their age group. In conclusion, our outcome measures address the effects of OPD on nutritional status, growth patterns, and health problems like gastroesophageal reflux (GER) and respiratory infections, explaining how swallowing difficulties can lead to additional health issues in children with CP. By organizing our evaluation using the PICO framework, we aimed to fully grasp the intricate nature of OPD in CP patients, highlighting its frequency, variations, and health effects to assist healthcare providers and caregivers in conducting focused evaluations and treatments to enhance nutritional and health results in this at-risk group.

### Inclusion criteria

This systematic review focused on studies targeting individuals with CP who had been diagnosed with OPD or similar swallowing disorders. Studies were considered eligible if they provided quantitative or qualitative data on the prevalence, types, or assessment methods of OPD in this population. Specifically, eligible studies are to those that explicitly included individuals with CP and reported swallowing assessments based on standardized diagnostic instruments such as videofluoroscopic swallowing studies (VFSS), fiberoptic endoscopic evaluation of swallowing (FEES), or validated clinical tools designed for dysphagia assessment. To ensure comprehensive coverage, we included studies examining the relationship between swallowing difficulties and nutritional health indicators, such as eating habits, growth patterns, and associated health conditions like GERD or respiratory complications. Only research articles published in peer-reviewed journals were considered, with no restrictions on the date of publication. Furthermore, the review exclusively included studies conducted on children with CP, focusing solely on human populations. Articles had to be published in English to be eligible.

### Exclusion criteria

Studies not centered on individuals with CP or lacking a definitive diagnosis of OPD were omitted. The omission also included articles that did not evaluate or discuss swallowing disorders in individuals with different neurological conditions, or those that did not examine or consider oral phase dysphagia with regard to nutritional status. Moreover, we excluded non-English publications or those without peer-review status, to maintain the credibility and dependability of the results. Exclusions were made for studies that focused mainly on surgical or invasive procedures and lacked relevant data on swallowing assessments or nutritional outcomes, as our review centers on non-invasive assessment techniques and their impacts on health. Systematic, integrated, or narrative reviews were also excluded; however, their reference lists were reviewed and included when relevant. Furthermore, diagnostic tools were excluded if they were found to overlap significantly with other included tools in terms of functionality, lacked adequate validation in clinical or research environments, or if there was insufficient evidence supporting their use specifically for the evaluation of oral phase dysphagia. Tools not directly addressing swallowing function or those designed for broader assessments unrelated to OPD were also omitted. This approach ensures that the included tools are both highly specific and clinically relevant for the target population. Finally, we did not include conference abstracts, opinion pieces, and editorials because they lack the necessary level of detail for a systematic review.

## Results

### Quality of included studies—Risk of bias

**Fig. 2:**
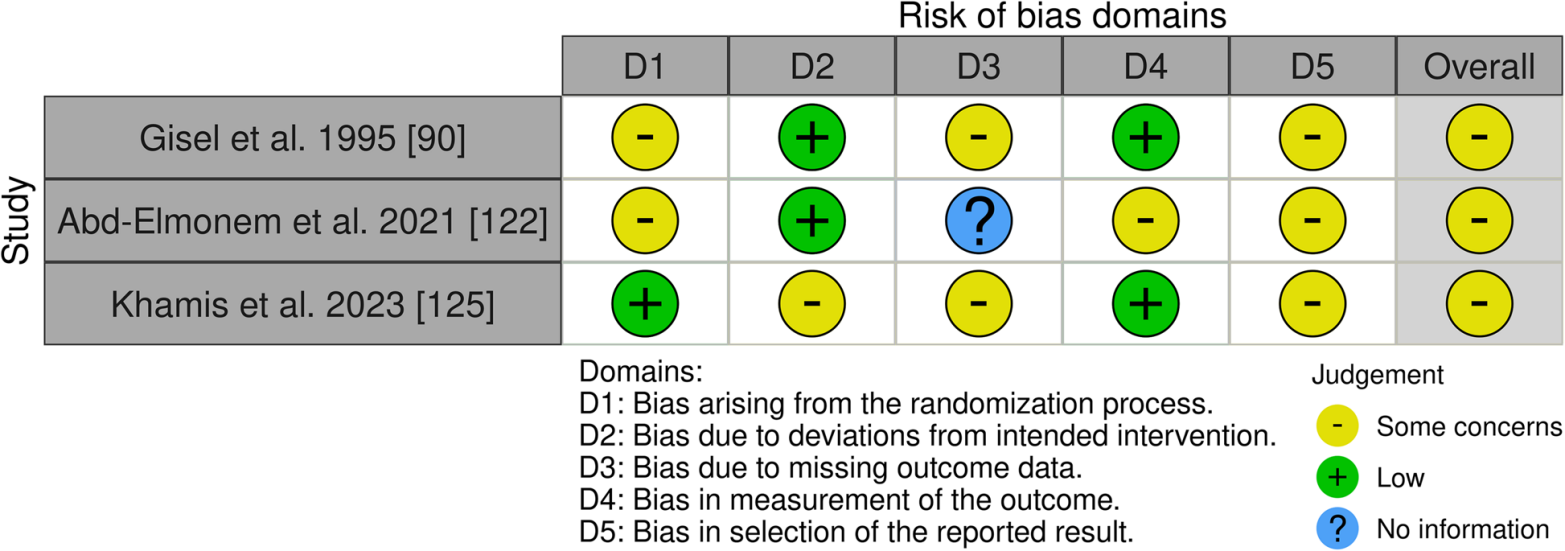
Risk of Bias (RoB) of included RCT studies

We assessed the risk of bias using appropriate tools based on the design of the included studies [83–123]. Of the forty-one studies, three were a randomized controlled trial (RCT) [88, 120, 123]. For this one, we used the updated Cochrane Risk of Bias (RoB 2) tool, which covers five domains: i) bias arising from the randomization process, ii) bias due to deviations from the intended intervention, iii) bias due to missing data on the results, iv) bias in the measurement of the outcome and v) bias in the selection of the reported result (Fig. 2) [124]. Our assessment identified mainly some concerns about the overall risk of bias and in particular in the selection of the reported result (D5). Gisel et al. [88], and Abd-Elmonem et al. [120], expressed some concern about the risk of bias in the randomization process (D1). This could introduce selection bias, potentially leading to imbalances in baseline characteristics between intervention groups. It could confound the observed effects and make it difficult to attribute changes solely to the intervention. On the other hand, Gisel et al. [88], and Khamis et al. [123] showed some concern risk in D3 (missing outcome data). The majority of the studies presented a low risk in the outcome measure (D4) and in the deviations from intended interventions (D2). Overall, while the RoB 2 assessment suggests reasonably sound methodology for these three studies, the identified biases, particularly in randomization, missing data, and reporting, warrant careful consideration when interpreting the synthesized evidence. These biases could potentially inflate or deflate the observed effects and limit the generalizability of the findings.

**Fig. 3:**
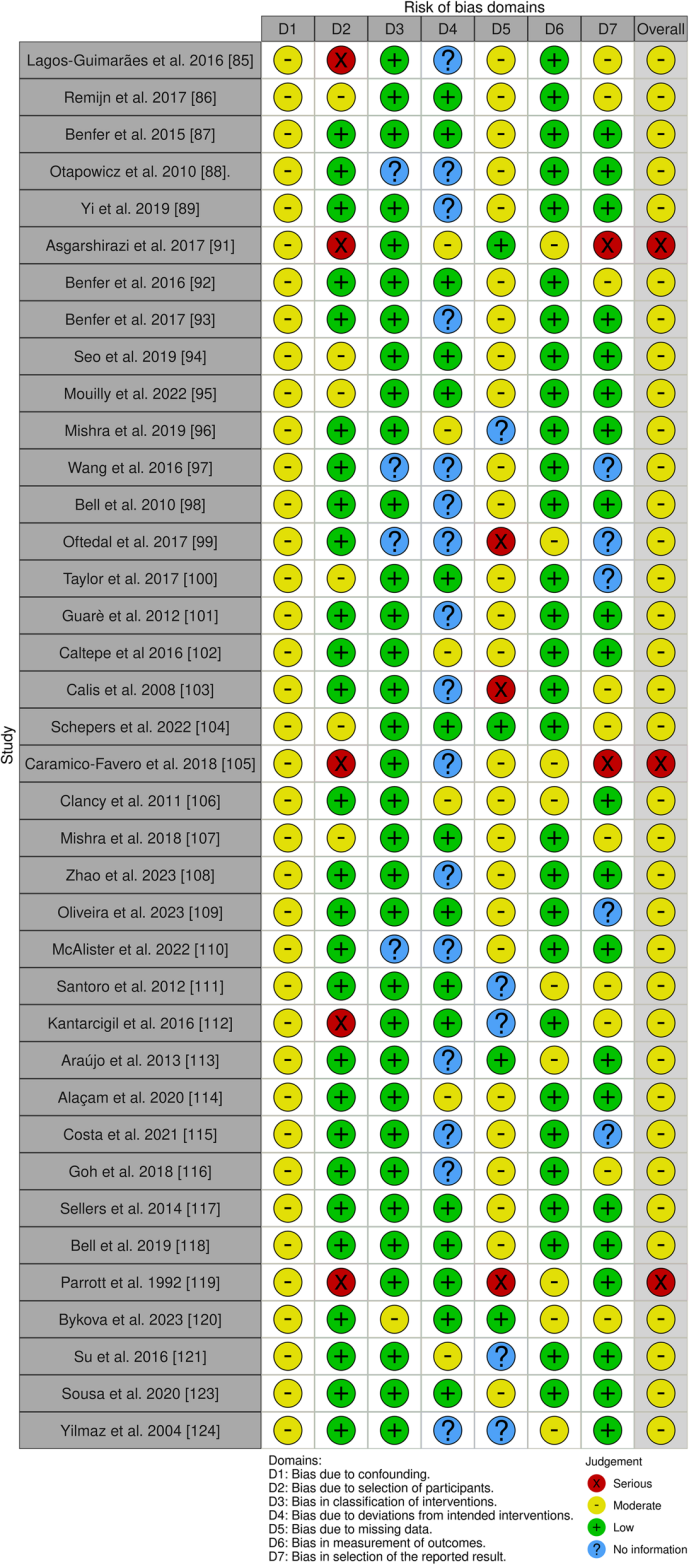
Cochrane Risk of Bias in Non-randomized Studies of Interventions (ROBINS-I)

For the thirty-eight [76, 80–83, 83, 84, 84, 85, 85, 86, 86, 87, 87–119, 121, 122] non-randomized studies we applied the ROBINS-I tool. ROBINS-I assesses bias in seven areas: i) bias due to confounding, ii) bias in participant selection, iii) bias in classification of interventions, iv) bias due to deviations from intended interventions, v) bias due to missing data, vi) bias in outcome measurement, and vii) bias in selection of the reported outcome (Fig. 3) [125]. The evaluation of bias with the ROBINS-I tool in different studies shows a generally moderate methodological standard, highlighting several areas of concern that may impact the accuracy of the results. For instance, bias in participant selection (D2) was a recurring issue in multiple studies, such as Lagos-Guimarães et al. [83], which showed a serious risk of bias in this domain. This could result in an unrepresentative sample that undermines the generalizability of the findings. Similarly, Remijn et al. [84] demonstrated moderate risks in both participant selection (D2) and confounding (D1), suggesting potential discrepancies in how baseline differences between intervention groups were managed. These biases may lead to distorted effect estimates, affecting the validity of their conclusions. Several studies, including those by Benfer et al. [85], and Benfer et al. [90], showed a minimal bias in various aspects. Nonetheless, they did highlight a moderate risk related to missing data (D5), which could impact the credibility of their results. Missing data, especially when systematic, could introduce imbalances or reduce the statistical power of the studies, thereby impacting the robustness of their findings. Conversely, concerns were significantly raised by Asgarshirazi et al. [89] regarding a study that faced a high risk of bias in participant selection (D2) and result reporting (D7), potentially compromising the reliability of its findings. This means that only certain results, perhaps those that are statistically significant or more favorable, are reported, while others are not. This can significantly distort the overall picture of the intervention's effects. Adding to the complexity of the evaluation, research conducted by Otapowicz et al. [86], Wang et al. [95], Oftedal et al. [97], and McAlister et al. 2022 [108] highlighted doubts concerning how interventions were classified (D3), as well as showing moderate/serious risks of data being absent (D5). This scenario highlights the difficulties in reaching firm conclusions from these inquiries. Furthermore, it is important to mention that many studies have shown moderate to severe problems in different areas, indicating systemic methodological issues that could have a significant effect on the accuracy and validity of the results. In summary, while these studies provide valuable insights, the identified biases, particularly in participant selection, confounding, missing data, and selective reporting, highlight the need for cautious interpretation of their results. These biases may potentially distort the observed effects, limiting the applicability of the findings to broader populations or clinical settings.

### Synthesis of evidence

In total, 1771 articles were found: 290 articles were removed due to duplication after screening; 56 articles were excluded because they were not published in English; 1171 articles were excluded based on title and abstract screening. Finally, 213 articles were removed based on screening for inadequate and untraceable study designs (as shown in Fig. 1). Forty-one research articles met the inclusion criteria and were therefore included in the review. These studies are summarized in Table 2.

**Table 2:** shows the main findings of the studies included in the review

**Author**	**Aim**	**Study Design/Intervention**	**Treatment Period**	**Sample Size**	**Sample Characteristics**	**Outcomes Measures**	**Main Findings**	**Statistical Analysis/Effects Size**
Khamis et al. 2023 [123]	To investigate if the babiEAT program is feasible and acceptable for infants at risk of CP compared to standard care	Randomized Controlled Trial	12 weeks	38 infants (19 per group)	*Age*: Mean age of 9.5 months (SD = 2.03) at randomization *Gender*: not Specificated	FIPQ, GAS, FSIS	The research emphasized that the babiEAT program was found to be feasible and acceptable in comparison to usual care	This pilot study did not calculate power for effect size because there was no available data, and a power calculation was not conducted
Abd-Elmonem et al. 2021 [120]	To assess whether a particular intervention can enhance oral motor skills in children with spastic CP	Randomized Controlled Trial	November 2018 to April 2020	Out of 70 children who signed up, 64 successfully finished the research, with 32 in each group	*Age*: 12 to 48 months *Gender*: both genders included	OMAS, SATco, GMFM-88	The participants experienced noticeable enhancements in oral motor skills and trunk control as a result of the intervention	The study reports an effect size of 0.75, determined using Cohen's d
Gisel et al. 1995 [88]	To evaluate how well oral sensorimotor therapy improves feeding abilities in children with CP	Randomized Controlled trial	Group 1 received 20 weeks of oral sensorimotor therapy, while Groups 2 and 3 underwent 10 weeks without formal therapy before receiving 10 weeks of the same treatment	27 children	*Age*: Mean age of children was 4.8 years (Group 1), 5.0 years (Group 2), and 5.4 years (Group 3) *Gender*: 14 boys and 13 girls	The amount of time spent chewing (in seconds) from when the food entered the mouth until the first swallow, along with an evaluation of functional feeding abilities using an adapted version of the Functional Feeding Assessment	After the treatment period, there were notable enhancements in the chewing duration of individuals in the aspirating group when consuming puree texture, suggesting that oral sensorimotor therapy could improve feeding efficiency. Groups that did not aspirate showed no substantial alterations in eating duration	Not Specificated
Lagos-Guimarães et al. 2016 [83]	To assess how the severity of dysphagia in children with CP is linked to factors like aspiration pneumonia	Cross-sectional study	September 2009 to December 2012	103 children	*Age*: 0 to 14 years (average age 4.26 years, standard deviation 4.18) *Gender*: 46 girls (44.66%) and 57 boys (55.34%)	Videofluoroscopic swallowing study and the Videofluoroscopic Dysphagia Severity Classification	Mild dysphagia was the most frequent level of severity, with many children experiencing severe dysphagia relying solely on oral nutrition support. There was a strong correlation noted between age and tracheal aspiration, with severe dysphagia cases showing a higher incidence of silent aspiration	Statistical analysis employed the mean to conduct descriptive statistics and utilized chi-square tests to evaluate relationships between variables, with a significance level of p < 0.05
Yilmaz et al. 2004 [122]	To assess eating ability and related challenges in individuals with spastic CP who are undergoing feeding intervention	Prospective Observational Study	Not Specificated	23 children and young adults	*Age*: between 4 and 25 years (mean age 10.5 ± 5.9) *Gender*: 14 males and 9 females	Functional Feeding Assessment Subscale of the Multidisciplinary Feeding Profile,	Most participants demonstrated varying levels of difficulty in feeding abilities, with 69% of those classified as having moderate to severe impairments experiencing problems with swallowing. Saliva leakage was seen in 74% of the patients, impacting their ability to eat, especially when using a straw or swallowing. Younger children had more trouble spoon feeding and using a straw compared to older children	The statistical analysis concentrated on evaluating variations in feeding performance scores among children with and without drooling. In particular, the Mann–Whitney U-test showed statistically significant differences (P < 0.05), suggesting that patients who drool had notably lower scores in these aspects compared to patients who do not drool
Remijn et al. 2017 [84]	To assess the oral-motor abilities of children with CP in relation to TD children while eating a crispy cookie, using different assessment methods	Comparative Observational Study	Not Specificated	There is a total of 22 children, 8 of whom have CP and the other 14 are TD children	*Age*: CP group: Mean age 9 years 8 months (range 7 years 3 months to 11 years 5 months). TD group: Mean age 9 years 1 month (range 5 years 5 months to 11 years 10 months) *Gender*: CP group: 3 boys. TD group: 6 boys	MOE	Compared to the TD group, the CP group showed decreased lateral and vertical tongue movement along with a lower frequency of tongue movements. The CP group exhibited longer chewing cycle durations and greater variability in their chewing cycle durations. In addition, CP children's MOE scores showed decreased oral-motor abilities, especially in swallowing and fluency/coordination when compared to TD children	Statistics were used to describe the outcome measures, including medians, means, and confidence intervals, while independent t-tests were used to compare the groups. Reliability within and between raters was assessed through intraclass correlation coefficient, with a value exceeding 0.80 suggesting robust agreement in manually tracing tongue contours and reliable replication in mandible movements
Benfer et al. 2015 [85]	To evaluate clinical indications indicating possible swallowing difficulties during the pharyngeal phase in young children with CP and to verify these indications through direct evaluations and feedback from parents	Cross sectional study	Between April 2009 and March 2013	A total of 130 children with CP	*Age*: 18–36 months *Gender*: 21 males (52.5%) in the TD group	GMFCS and Clinical signs of pharyngeal phase impairment	37.5% of children with TD showed clinical signs, with notable variations in signs between children with CP at GMFCS IV and V compared to those in the TD group	OR = 1.4 for the entire model and 3.9 is the equivalent of three point nine. An OR of 3.9 suggests that children with lower gross motor function are more likely to show clinical signs when comparing level V to level I
Otapowicz et al. 2010 [86]	To evaluate early swallowing issues in children with ICP and how they relate to cognitive abilities and the specific type of ICP	Cross Sectional Study	Not Specificated	67 children	*Age*: 3 to 17 years, with a mean age of 8.5 years (SD = 4.3) *Gender*: 39 boys (58.2%) and 28 girls (41.8%)	Maternal interviews, dysarthria Scale, speech apparatus and swallowing functioning assessed by a logopaedist	The research discovered that 69% of kids experienced issues with sucking and swallowing, while 61% were currently struggling mainly during the oral phase. The seriousness of swallowing problems was linked strongly to the specific type of ICP and the extent of cognitive difficulties, as dysarthria was found in every child with oral phase difficulties	The research used percentage calculations and the chi-square test for statistical analysis, establishing significance at P < 0.05. The study found a significant link between the seriousness of swallowing problems and the specific type of CP, highlighting that children with tetraplegia showed the most severe oropharyngeal phase issues (p < 0.044)
Yi et al. 2019 [87]	To compare the effects of dysphagia symptoms on quality of life in adults with CP versus healthy individuals	Cross Sectional study	Between January 2018 and June 2018	117 adults with CP and a group of healthy adults	*Age*: 20 years and older *Gender*: both male and female	MMSE, SWAL-QOL, FOIS	Adults with CP showed notably poorer quality of life related to swallowing issues compared to healthy adults, with choking on food being the most common symptom mentioned. Furthermore, predictors of the SWAL-QOL composite score included factors such as FOIS level and age	The effect size in SWAL-QOL scores between healthy adults and adults with CP was found to be highly significant, as healthy individuals had an average score of 97.39 while those with CP scored 60.60 (P < 0.001)
McAlister et al. 2022 [108]	The study sought to evaluate the eating and drinking skills of adults with CP and determine the features linked to dysphagia	Cross Sectional study	Data was gathered from grown-ups with CP enrolled in the CP monitoring program and database starting in 2016 up to June 2021	2,035 adults	*Age*: 18 to 78 years *Gender*: 1,122 males (55.1%) and 913 females (44.9%)	EDACS	Over half of the individuals (52.5%) were able to consume food and beverages in a safe and effective manner (EDACS level I). Nevertheless, 32.4% were grouped in EDACS categories III to V, signaling restricted safety during meals and beverages consumption. Moreover, elderly individuals and individuals with more advanced types of CP experienced increased difficulties with regards to consuming food and beverages	Simple and multivariable linear regression models were used in this study's statistical analysis to examine the connections between independent variables and weight in kilograms. Findings were displayed as unadjusted and adjusted unstandardized coefficients, along with 95% confidence intervals. The models' goodness-of-fit was assessed with R^2^, showing the proportion of weight variance explained by all independent variables
Asgarshirazi et al. 2017 [89]	To examine feeding disorders and their connection with GERD and oropharyngeal dysfunction, a study will be conducted on children with CP who are facing feeding issues like choking, repeated pneumonia, and inadequate weight gain	Cross Sectional study	6 months	50 children	*Age*: The average age of the participants was 6.28 years, ranging from 2 to 12 years *Gender*: 56% of the patients were boys	Fluoroscopy was used for a barium swallow, Upper endoscopy was performed with esophageal biopsies, Evaluation of choking episodes, respiratory infections needing hospitalization, and weight gain after six months of therapy were assessed	The research discovered that 82% of individuals had swallowing issues and 66% had GERD, with notable enhancements in incidents of choking (reduced by 76%) and pneumonia (reduced by 66%) after half a year of therapy, but only 46% of kids saw an increase in weight. Moreover, there was a strong correlation between severe gross motor function and cognitive delays and increased prevalence of oral-motor incoordination and GERD	The study used Chi-square tests for statistical analysis to compare the occurrence of feeding disorders and GERD in children with CP
Benfer et al. 2016 [90]	To examine how common OPD is in preschool-aged children with CP and how it relates to their health results	Longitudinal Cohort Study	April 2009 to April 2013	53 children	*Age*: Aged 22.9 months (SD = 2.9) *Gender*: not Specificated	DDS, Schedule for Oral Motor Assessment, and Pre Speech Assessment Scale	The frequency of OPD dropped from 62 to 59% as time passed. 30% of children showed enhancements in their feeding skills, with gross motor function being the sole consistent risk factor for delayed development in OPD across evaluations	Effect size was evaluated through different statistical approaches, such as McNemar’s Test for binary results, the Wilcoxon Matched Pairs Test for ordinal information, and paired T-tests for continuous factors. The reliability of the DSS was confirmed with a strong agreement rate of 90% and a kappa value of 0.8 (p < 0.001), showing significant consistency
Benfer et al. 2017 [91]	To assess OPD in young children with CP during their preschool years	Longitudinal Study	From April 2009 to March 2015	179 children took part, and initial findings from 53 children were disclosed	*Age*: Children aged 18 to 60 months at assessment *Gender*: not Specificated	DDS, EDACS, GMFCS, MACS	The research discovered a decrease in oral feeding problems in children with CP from 79.7% at 18–24 months to 43.5% at 60 months, with notable links between OPD results and GMFCS stages	OR were used to determine the impact of GMFCS and age on OPD outcomes. The results showed a decrease in OPD prevalence with increasing age (OR = 0.92) and a strong correlation with GMFCS levels (OR = 6.2)
Seo et al. 2019 [92]	To assess the swallowing ability and its correlation with cervical dystonia and gross motor function in adults with DCP using clinical evaluations and VFSS	Observational Study	August 2014 to March 2017	17 participants	*Age*: Age ranged from 34.9 to 59.0 years (mean age 47.7 ± 6.3 years) *Gender*: 8 men and 9 women	CDS, VDS, PAS, GMFCS, TWSTRS	The research discovered common irregularities in swallowing abilities among subjects, mainly including insufficient chewing and limited tongue mobility. A strong relationship was found between VDS total scores and TWSTRS scores, suggesting a connection between difficulty swallowing and the severity of cervical dystonia	The impact sizes were evaluated by testing the reliability within and between raters, resulting in highly impressive intraclass correlation coefficients for both the VDS and PAS, with values of 0.977 and 0.990, respectively
Mouilly et al. 2022 [93]	The research sought to examine the physical measurements and swallowing issues in kids with CP, while also evaluating their dental health	Cross Sectional Study	Not Specificated	65 patients	*Age*: 2 to 17 years, with a median of 9.25 years *Gender*: Predominantly female (52.3%)	Measurements of weight and height, evaluation of GMFCS, examination of swallowing problems, and assessment of oral health	It was revealed that 64.6% of individuals suffering from CP showed signs of swallowing problems, and 52.3% experienced constipation, with 95% needing modified diets. Furthermore, 26.2% of patients had oral-facial malformations, resulting in notable variations in nutritional indices due to the existence of these malformations and swallowing difficulties	Effect sizes were computed to evaluate how oral-facial malformations and swallowing disorders affect the nutritional indices of children with CP, showing notable discrepancies in weight-for-age z-scores (p = 0.001) and height-for-age z-scores (p = 0.0001)
Mishra et al. 2019 [94]	To assess the feeding and swallowing abilities, swallowing physiology, and voluntary cough in children with speech sound disorders in comparison to typically developing children	Comparative study	Not Specificated	21 children (11 with SCP and 10 TDC)	*Age*: 4–11 years *Gender*: not Specificated	DDS, sEMS, respiratory Inductance Plethysmography for respiratory-swallow coordination, voluntary cough assessment	Children with SCP had notably more severe feeding and swallowing issues than TDC, needing to use a higher percentage of maximum amplitude for swallowing puree and solid foods. TDC did not show any signs of aspiration, whereas three children with SCP coughed during the assessment	Effect sizes were computed for relevant measures to assess the extent of variations among groups. Statistical analysis was performed through nonparametric methods because of the data's non-normal distribution and limited power, evaluating intra- and interrater reliability using intraclass correlation coefficients and Cohen's kappa. The significance level's alpha value was established at p < 0.05
Wang et al. 2016 [95]	To assess the development and dietary health of children with CP residing in Chengdu	Cross Sectional Study	February to April 2013	377 children with CP aged between 2 to 18 years	*Age*: Mean age of 7.9 years (± 3.5 years) *Gender*: 53.6% male, 46.4% female	GMFCS, MACS, anthropometric measures (height, weight)	The research discovered that 42.4% of kids with CP were experiencing stunted growth, while 12.7% were considered underweight, with a notably higher occurrence of these issues among those identified as GMFCS IV-V in comparison to GMFCS I-II. Moreover, 59.9% of the children were classified as having a normal weight based on BMI evaluations, showing that motor function severity significantly influences nutritional status	Different statistical measures, like average and standard deviation, were used to assess effect sizes for continuous variables such as age, weight Z scores, and height Z scores. The Mann–Whitney Test and Kruskal–Wallis test were employed to identify variations in growth and nutritional status among gender and age groups, while chi-square tests were used for categorical variables, with a significance level of P < 0.05 for all analyses
Bell et al. 2010 [96]	To explore how linear growth, body composition, oral motor and feeding issues, dietary intake, sedentary behavior, and their effects on health outcomes, healthcare needs, participation, and quality of life in young children with CP connect	Prospective Longitudinal cohort study	Evaluations will be carried out at three different time points: between 17 and 25 months, at 36 ± 1 months, and at 60 ± 1 months corrected age	The objective of the research is to enroll 240 kids in total	Participants in the study will consist of children with CP who were born from September 1, 2006 to December 31, 2009, with a variety of ages and genders expected	GMFM-66 and GMFCS	This research will offer the initial longitudinal analysis on how functional achievement relates to changeable lifestyle elements (eating habits and sedentary habits) and how they affect growth, body structure, and nutritional status in young children with CP regardless of their functional capacity	Descriptive statistics will outline participant traits, while inferential statistics, including mixed-effects models, will assess variations over time and distinctions among groups categorized by the GMFCS
Sousa et al. 2020 [121]	To examine the relationship between the nutritional status of children and adolescents with spastic quadriplegic CP and factors related to their diet and feeding method	Cross Sectional Study	Between July 2016 and January 2017	28 patients	*Age*: Patients aged ≤ 13 years old *Gender*: not Specificated	Nutritional status evaluation (Body mass index, weight, height), Dietary complications (feeding route, type of diet), 24-h dietary recall	The research revealed that 75% of patients opted for alternative methods of feeding, with 57% being deemed as having a normal nutritional status. There was a direct correlation discovered between the diet ingested and nutritional status, with no correlation found between complications and nutritional status	Effect sizes was measured through correlations linking caloric intake and nutritional status, with noted results (r = 0.48, p = 0.01), suggesting a favorable connection between dietary quality and nutritional results
Oftedal et al. 2017 [97]	To explore how WZ, FFM, and %BF interact with lifestyle factors in children with CP of all GMFCS levels over time	Longitudinal Cohort Study	From April 2009 to March 2015, evaluations were conducted every 12–18 months	161 children diagnosed with CP, undergoing a total of 364 evaluations	*Age*: Age range of participants was 18–60 months (mean age: 2.8 years); GMFCS distribution: I (48%), II (11%), III (15%), IV (11%), V (15%) *Gender*: 61% were boys	WZ, FFM, GMFCS, %BF, HPA	Changes in body composition were observed in young children with CP at different GMFCS levels, showing varying body composition traits; higher energy intake and HPA levels were linked to increased FFM in GMFCS group I kids, emphasizing the role of physical abilities and lifestyle in body composition	The statistical analysis involved using mixed-effects linear models to explore age-related changes in weight-for-age z scores, BMI-for-age z scores, fat-free mass, and percentage body fat
Taylor et al. 2017 [98]	To evaluate the efficacy of behavior analytic interventions when it comes to managing feeding challenges and reliance on feeding tubes in children diagnosed with autism and CP	Comparative Study	From 2003 to 2013	58 children	*Age*: 1 to 12 years, with a mean age of 69.53 months (SD = 30.69) *Gender*: 60% (35 children) were male	Grams of food consumed during meal blocks, frequency of food refusal and negative vocalizations, CEBI, and caregiver satisfaction regarding program effectiveness and goals met	The research discovered that both interventions of applied behavior analysis led to a significant enhancement in feeding abilities of children with autism and CP, with functional analysis showing positive results for 78% of the children. Furthermore, both interventions showed success in increasing parental involvement, emphasizing the significance of family participation in the treatment procedure	The examination showed important overall impacts for time on different measures, such as grams consumed, refusal actions, and negative vocalizations. Significantly, the mixed model repeated measures showed significant enhancements in the amount of food consumed orally, displaying an average increase of 121.91 g (p < 0.001) from the initial caregiver baseline to the last treatment session, resulting in a substantial effect size (partial η^2^ = 0.484)
Guarè et al. 2012 [99]	To assess the occurrence of GERD and its link with dental erosion, dietary intake, gastrointestinal symptoms, bruxism, and salivary flow rate in children with CP	Cross Sectional Study	Not Specificated	46 children	*Age*: 3 to 13 years *Gender*: both genders included	Presence of dental erosion, dietary habits (acid drink consumption), gastrointestinal symptoms (regurgitation and heartburn), presence of bruxism, salivary flow rate (measured in ml/min)	The research discovered that kids with CP and GERD had a much higher occurrence of dental erosion than those without GERD, and dental erosion was linked to GERD on its own. Moreover, quadriplegic individuals with GERD were found to be at higher risk for oral disease	Effect sizes were computed using odds ratios to assess the relationship between GERD and dental erosion in children with CP. Statistical tests for normality included Shapiro–Wilk and Kolmogorov–Smirnov tests, and mean comparisons were conducted using Mann–Whitney tests
Caltepe et al. 2016 [100]	To study GER in children with CP by utilizing a combination of MII-pH monitoring	Prospective Study	24-h combined MII-pH monitoring	30 kids were registered, and enough information was gathered from 29 individuals	*Age*: Mean age of 4.0 ± 2.3 years *Gender*: 16 females (55.2%) and 13 males (44.8%)	MII-pH	In total, there were 3,899 recorded reflux episodes, with 29% identified as acid, 60% as weakly acid, and 11% as alkaline. The research found that GER is prevalent in children with CP, predominantly consisting of non-acidic episodes, and that pH testing alone would not detect 70% of these episodes	The impact size was determined through the average and standard deviation of reflux occurrences among the subjects in the research, indicating a notable disparity in weakly acidic reflux frequency versus acidic reflux (p < 0.010)
Calis et al. 2008 [101]	To evaluate the clinical signs and seriousness of trouble swallowing in a typical group of kids with severe widespread CP and mental disability	Longitudinal Study	Not Specificated	166 children	*Age*: Mean age was 9 years 4 months, with a range from 2 years 1 month to 19 years 1 month *Gender*: 85 males and 81 females	DSS	The research discovered a high occurrence of dysphagia (99%) in kids with severe generalized CP and intellectual disability, with 76% showing moderate to severe dysphagia and 15% categorized as profound dysphagia. The severity of dysphagia was found to have a positive correlation with the severity of motor impairment and, surprisingly, was linked to a higher weight for height, suggesting that parents may be underestimating feeding issues	Various statistical tests, such as analysis of variance, Mann–Whitney U test (z-score), and Spearman's rank correlation, were used to assess relationships between general child characteristics, DDS Part 1, and parental questionnaire items with the dysphagia severity scale
Schepers et al. 2022 [102]	To compare dysphagia limits in children with CP based on EDACS level, gender, and age with those of typically developing children	Comparative Study	Not Specificated	77 children	*Age*: Mean age of 7 years and 6 months (SD 2 years 2 months; age range 4–12 years) *Gender*: 54 males and 23 females	Maximum Volume Water Swallow Test	Children diagnosed with CP had a much lower median dysphagia limit (3.0 mL) than children who typically develop (22.0 mL). The severity of dysphagia decreased as the EDACS level increased, accounting for 55% of the variability in the dysphagia limit	The research employed the Mann–Whitney U test to examine dysphagia thresholds in children with CP and normally developing peers, resulting in a statistically significant outcome (U = 1279.5, p < 0.001, r = 0.65), showing a substantial impact
Caramico-Favero et al. 2018 [103]	To examine the link between eating habits, nutritional health, and digestive issues in kids with CP	Cross Sectional Study	Not Specificated	40 children	*Age*: 4 to 10 years (mean age 6.7 ± 2.4 years) *Gender*: 23 (57.5%) male and 17 (42.5%) female	Anthropometric measurements, evaluation of typical household food consumption, assessment of GI symptoms, and testing blood for hemoglobin and ferritin levels are methods used for dietary assessment	Children with CP showed noticeable stunted growth, with a median height-for-age Z-score of −4.05, and a high occurrence of gastrointestinal issues: 82.5% dealt with difficulty swallowing, 40% had GER, and 60% suffered from constipation, resulting in reduced energy and fluid consumption for those with swallowing difficulties	Both parametric and non-parametric tests were utilized for statistical analysis based on the data distribution. Notable disparities were discovered in height-for-age Z-scores in relation to weight-for-age and weight-for-height, with a p-value below 0.05 demonstrating a considerable deficiency in growth among the subjects
Clancy et al. 2011 [104]	To investigate how feeding patterns vary among children with CP with different levels of oral motor impairment and track changes in feeding habits over time across severity levels	Longitudinal Observational Study	30 months, gathering data every 6 months	23 children	*Age*: Mean age of 4.53 years (SD = 0.41 years) *Gender*: 9 females and 14 males	FSQ, discussing tube feeding, thickened liquids, unique feeding methods, feeding therapy, studies on swallowing, instances of choking, and coughing	Most feeding variables showed notable disparities between severity groups, except for choking and coughing, where individuals with severe cases experienced the greatest challenges. Over time, the "within normal limits" group was the only one to experience a notable change in coughing, with other factors staying consistent among severity groups	Chi-square tests were used to analyze the effect size, which aimed to explore variations in feeding and swallowing behaviors across three severity groups of children with CP. Significant variances were noted in tube feeding (χ^2^ = 11.32, p = .003), thickened liquids (χ^2^ = 6.16, p = .046), special feeding techniques (χ^2^ = 12.68, p = .002), feeding therapy (χ^2^ = 7.45, p = .024), and swallow studies (χ^2^ = 8.63, p = .013)
Mishra et al. 2018 [105]	To assess the dependability of fresh methods to measure mealtime length and explore their connection with the clinical feeding/swallowing abilities in children with SCP	Observational Study	Information was gathered at two summer camps in the years 2014 and 2015	17 children	*Age*: Range from 5 years and 1 month to 17 years and 6 months *Gender*: 9 boys and 8 girls	DDS	The recently created measures for mealtime duration showed high reliability, with mealtime duration being positively correlated with DDS scores. Kids with one-sided brain involvement showed improved feeding/swallowing ability, shorter meal times, and lower DDS scores in comparison to those with brain involvement on both sides	Eta squared values were used to calculate effect sizes for the Kruskal–Wallis H test, showing significant differences in mealtime durations and DDS total scores, which further supported the study's results
Zhao et al. 2023 [106]	To discuss the nutritional status and features of children with CP and to investigate the connection between CP severity and nutritional status	Cross Sectional Study	July 2020 to June 2021	1151 participants	*Age*: Children aged 1–18 years *Gender*: 49 males and 402 females	GMFCS, EDACS, SGNA, social life ability scale, and serum blood indicators	50.8% of children were found to be experiencing undernutrition. Children with GMFCS and EDACS levels IV and V had a noticeably greater chance of experiencing moderate to severe undernutrition than those in levels I-III. There were no notable variations in blood markers (aside from serum 25-hydroxyvitamin D) among different nutrition status categories	OR and 95% confidence intervals were used to determine effect sizes for examining the connection between nutritional status and motor function as categorized by the GMFCS and EDACS
Oliveira et al. 2023 [107]	To evaluate the nutritional condition of adult individuals with CP and swallowing difficulties who have stayed in a long-term care facility for more than a decade	Prospective Cohort Study	Between December 2015 and December 2016	56 patients	*Age*: 25 to 71 years (Mean: 44 ± 12 years) *Gender*: 27 men (48%) and 29 women (52%)	Body Mass Index, PARD, FOIS	There were no notable differences in weight, nutritional status, diet consistency, dysphagia, or functional eating levels between the two evaluation periods. Nevertheless, greater dysphagia severity and strict dietary restrictions were linked to worse nutritional status	Effect sizes were calculated using the Wilcoxon and McNemar tests for continuous variables, and Fisher’s exact test was used for categorical variables. Findings were shown as averages, deviations, and middle values for continuous factors, and as total occurrences for categorical factors, with significance indicated at P < 0.05
Santoro et al. 2012 [109]	To assess clinical markers and severity of feeding difficulties in kids with neurodevelopmental conditions, especially CP, using a thorough multidisciplinary evaluation	Observational Study	Not Specificated	40 children	*Age*: Mean age of 38 months (range, 4–136 months) *Gender*: 26 boys and 14 girls	GMFCS, clinical evaluation with a mealtime observation, and a modified barium swallow examination	All participants had feeding issues, and 37 out of 40 were found to have dysphagia. Of these, a high number had severe bilateral CP, exhibiting decreased swallowing frequency, sucking-swallowing coordination issues, and diverse oral sensitivity abnormalities. Moreover, 20% of the individuals also showed redness in the back of the throat, indicating stomach acid coming up the esophagus which was verified through a barium swallow in all potential instances	Statistical analysis showed that over 90% of the participants had adequate sucking abilities, with 50% showing decreased swallowing frequency, and more than two-thirds demonstrating sucking-swallowing coordination issues, indicating significant oral motor function challenges within the group. Data was gathered using a thorough assessment protocol, and descriptive statistics were used to summarize the clinical characteristics and feeding problems seen in the participants
Kantarcigil et al. 2016 [110]	Assessing the trustworthiness of a telehealth model for standardized clinical evaluations of swallowing in children with CP, by comparing remote evaluations to in-person assessments	Prospective Cohort Study	Observation and evaluation conducted for three days in a row during lunch periods	19 children	*Age*: Mean age of 11.6 years (range: 6.9–17.5 years) *Gender*: 12 males and 7 females	DSS, DMSS	Significant agreement was observed between in-person and asynchronous online assessments by the same evaluator for five out of the seven DDS Part 2 variables	The weighted kappa coefficient (KW) was used to evaluate the effect size, with values ranging from 0 to 1, showing the level of agreement between assessments. Intra-rater agreement among the same raters was high to very high (KW = 0.64–1), whereas inter-rater agreement among different evaluators showed high agreement levels (KW = 0.62–0.86)
Araújo et al. 2013 [111]	To evaluate the nutritional health of children with CP, we will compare CP-specific growth charts with general growth charts and investigate how growth measurements are related to digestive issues like dysphagia, constipation, and respiratory infections	Cross Sectional Study	Data was gathered from individuals who were hospitalized at a rehab facility between March 2001 and March 2007	187 individuals	*Age*: Mean age of 5.6 years (± 3.5 years) *Gender*: 58% were male	Anthropometric data (weight, height), assessment of dysphagia, constipation, and recurrent respiratory infections	The research discovered a notable difference in growth curves between CP and general pediatric patients, showing that standard curves tend to overestimate malnutrition in CP individuals. Furthermore, the majority of people with dysphagia, respiratory infections, and constipation had weights below the 50th percentile, showing a weak association between CP-specific and general growth standards	The effect size and statistical analysis were assessed mainly using descriptive statistics and inferential tests, such as the chi-squared test and the Kruskal–Wallis H test, to compare categorical variables. The weighted kappa coefficient was used to evaluate the consistency of agreement among various reference growth curves, with a significance level of 5% being established. The research discovered a significant occurrence of malnutrition and weight disparities below the 50th percentile in both the CP and CDC growth charts, emphasizing substantial variations in the gathered anthropometric information
Alaçam et al. 2020 [112]	To assess the presence of OFD in Turkish children with CP using the NOT-S and to contrast results with those of a healthy control group	Cross Sectional Study	August 2017 to September 2017	84 children in total (42 with CP and 42 healthy controls)	*Age*: Children aged 3–16 years *Gender*: both groups included 20 boys and 22 girls each	NOT-S	Children diagnosed with CP showed higher scores in NOT-S interviews and clinical examination, which pointed to more frequent OFD in comparison to healthy individuals. The majority of dysfunctions were found in the areas of facial expression (55.9%), chewing/swallowing (52.4%), and sensory function (47.6%)	The effect size was calculated by comparing the NOT-S screening test scores between children with CP and healthy controls using the Mann–Whitney U test for data that is not normally distributed. The findings showed a notable increase in the total NOT-S score among the CP group (p < 0.001), suggesting a prominent presence of orofacial dysfunction
Costa et al. 2021 [113]	To evaluate how common OD, malnutrition, dehydration, and oral health issues are among students at a special needs school with severe neurological disabilities including CP	Cross Sectional Study	February 1, 2019, to May 30, 2019	33 students	*Age*: Mean age of 13.3 years *Gender*: 36.4% female	GMFCS, V-VST, EDACS, OHIS, evaluation of nutrition and hydration through measuring body size, electrical impedance, and food intake documentation	Every student diagnosed with OD demonstrated compromised safety while swallowing, with 90.6% exhibiting this issue. Additionally, 68.7% were categorized as levels II-III of EDACS, and 31.3% needed PEG feeding. Furthermore, 89.3% experienced chronic malnutrition, 21.4% were acutely malnourished, 70% had dehydration at a cellular level, and 83.9% showed signs of inadequate oral health	Effect sizes were computed in this study to gauge the size of observed effects in different clinical and functional evaluations, including GMFCS levels, swallowing function, and masticatory capacity. A statistical analysis was carried out using confidence intervals, p-values, and reliability metrics, such as the kappa value, to evaluate differences and relationships between important variables
Goh et al. 2018 [114]	To explore how different classification systems evaluating the seriousness of OD and communication function relate to other functional profiles in children with CP	Cross Sectional Study	From March 2016 to February 2017	151 children with CP	*Age*: Mean age of 6.11 years (SD 3.42, range 3–18 years) *Gender*: non Specificated	EDACS, FOIS, SFS, FILS, CFCS, VSS, PEDI, GMFCS, MACS	15.2% of children were found to have high levels of dysphagia (EDACS level III-V). There were significant correlations between EDACS and dysphagia scales, as well as with MACS, CFCS, and VSS. Inadequate performance in EDACS correlated with deficiencies in gross motor skills and communication abilities	Effect sizes were calculated using Spearman correlation coefficients, with associations classified as very strong (≥ 0.80), strong (0.80–0.60), moderate (0.60–0.40), weak (0.40–0.20), and very weak (≪ 0.20). For all tests, a significance level of p < 0.05 was utilized, categorizing functional measures into two groups (good and poor functioning) for regression examinations
Sellers et al. 2014 [115]	To create an accurate system for categorizing eating and drinking skills in individuals with CP and assess its consistency	Methodology quality study	Not Specificated	The research included 56 individuals in the nominal groups and 95 individuals in the Delphi survey, comprising a total of 100 children assessed by SaLTs and 48 children assessed by both SaLTs and parents	*Age*: from 4 to 22 years *Gender*: not Specificated	EDACS	The findings showed that the EDACS is a dependable and accurate method for categorizing how individuals with CP eat and drink. The level of complete agreement among SaLTs was 78%, while the agreement between SaLTs and parents was 58%. Disagreements were usually minimal, which demonstrates the classification system's reliability for both clinical and research purposes	Kappa coefficients and intraclass correlation coefficients were utilized in the research to assess inter-rater reliability for the EDACS classifications. The kappa values showed substantial agreement (0.72) between SaLTs and moderate agreement (0.45) between SaLTs and parents. Intraclass correlation coefficients indicated high consistency, with 0.93 between SaLT pairs and 0.86 between SaLTs and parents
Bell et al. 2019 [116]	To create and confirm a screening instrument for identifying feeding/swallowing issues and/or malnutrition in children diagnosed with CP	Cross Sectional Study	Between February 2017 and March 2018	89 children with CP	*Age*: Median age of 6 years (range 4 to 8 years, 11 months) *Gender*: 63 males and 26 females	EDACS, DDS, SGNA, VFSS	The ultimate 4-item screening tool showed great sensitivity and specificity, correctly detecting 81% of children with feeding/swallowing issues and 72% with undernutrition. It effectively identified every instance of extreme malnutrition and children categorized as EDACS level IV or V	The effect size in this study was calculated by determining the best diagnostic properties of each questionnaire item for assessing feeding and nutritional difficulties, and choosing those with the highest sensitivity and specificity. Statistical analysis included summarizing data using mean, standard deviation, frequency, and percentage, as well as conducting chi-square tests for associations
Parrott et al. 1992 [117]	To determine if the EDAT is as effective as a thorough clinical evaluation by a team of different specialists, in assessing feeding issues in children with CP	Comparative Study	The EDAT assessment takes 15–20 min for each child and is conducted in a setting familiar to them	18 children	*Age*: 3 years, 7 months to 15 years, with a mean of 7 years, 8 months *Gender*: not Specificated	EDAT	EDAT demonstrated high agreement (minimum 78%) with clinical evaluations for assessing the designated feeding abilities. EDAT was found to be a successful, non-invasive method for detecting feeding difficulties in different skills that were evaluated	The effect sizes of feeding skills, such as anticipation, intraoral sensory perception, oral-motor efficiency, and pharyngeal triggering, was calculated by comparing each child's results to norms of the same age
Bykova et al. 2023 [118]	To assess how well the EDACS can predict aspiration risk in children with CP, by comparing it to the PEDI-EAT-10. Another objective is to explore how aspiration and non-aspiration groups are related based on EDACS and PEDI-EAT-10 classifications	Prospective Study	Not Specificated	131 children	*Age*: Median age of 4.4 years *Gender*: 77 males and 54 females	EDACS and PEDI-EAT-10	The EDACS demonstrated good accuracy in detecting aspiration risk in children with CP, with 78% sensitivity and 92% specificity based on the PEDI-EAT-10 as a comparison. It is advised to use both EDACS and PEDI-EAT-10 together to help determine if further swallowing studies are needed	Effect size analysis demonstrated the effectiveness of EDACS in detecting risk of aspiration, boasting a high specificity of 92% and sensitivity of 78%, yielding an area under the curve of 0.892 on the receiver operating characteristic curve
Su et al. 2016 [119]	To assess how well the MASA predicts VFSS outcomes in children with CP and suspected aspiration	Observational Study	Not Specificated	16 children	*Age*: from 6 to 19 years *Gender*: 5 girls and 11 boys	MASA and FDS	There was no significant difference in MASA scores between those who aspirate and those who do not, or between silent and overt aspirators. Nevertheless, MASA demonstrated predictive capability for oral dysfunction but not for pharyngeal function in VFSS	The effect size analysis showed a significant relationship between the MASA and FDS scores, with a strong negative correlation found for the oral phase subtotal FDS scores as indicated by the Spearman correlation coefficient (rs = −0.713, p < 0.05)

Legend: *CP* cerebral palsy, *FIPQ* Feeding Intervention Preferences Questionnaire, *GAS* Goal Achievement Scaling, *FSIS* Feeding Swallowing Impact Scale, *OMAS* Oral Motor Assessment Scale, *SATCo* Segmental Assessment of Trunk Control, *GMFM-88* Gross Motor Function Measure-88, *TD* typically developing, *MOE* Mastication Observation Scale, *GMFCS* Gross Motor Function Classification System, *OR* Odds Ratio, *ICP* infantile cerebral palsy, *MMSE* Mini-Mental State Examination, *SWAL-QOL* swallowing-quality of life, *FOIS* Functional Oral Intake Scale, *EDACS* Eating and Drinking Ability Classification System, *GERD* gastro-esophageal reflux disease, *OPD* oropharyngeal dysphagia, *DDS* Dysphagia Disorders Survey, *MACS* Manual Ability Classification System, *DCP* dystonic cerebral palsy, *VFSS* videofluoroscopic swallowing studies, *CDS* Clinical Dysphagia Scale, *VDS* Videofluoroscopic Dysphagia Scale, *PAS* Penetration-Aspiration Scale, *TWSTRS* Toronto Western Spasmodic Torticollis Rating Scale, *TDC* typically developing controls, *sEMG* Surface electromyography, *WZ* weight-for-age z score, *FFM* fat-free mass, *%BF* percentage of body fat, *HPA* habitual physical activity, *CEBI* Children’s Eating Behavior Inventory, *GER* gastroesophageal reflux, *MII* multichannel intraluminal impedance, *FSQ* Parent-reported Feeding and Swallowing Questionnaire, *SGNA* Subjective Global Nutritional Assessment, *PARD* Dysphagia Risk Assessment Protocol, *DMSS* Dysphagia Management Staging Scale, *OFD* orofacial dysfunctions, *NOT-S* Nordic Orofacial Test-Screening, *V-VST* Volume Viscosity Swallowing Test, *OHIS* Oral Hygiene Index Simplified, *SFS* Swallow Function Scales, *FILS* Food Intake Level Scale, *CFCS* Communication Function Classification System, *VSS* Viking Speech Scale, *PEDI* Pediatric Evaluation of Disability Inventory, *SaLTs* speech and language therapists, *EDAT* Exeter Dysphagia Assessment Technique, *PEDI-EAT-10* Pediatric Eating Assessment Tool, *FDS* Functional Dysphagia Scale

CP does not have any cure, and only a few therapies assist in the management of the condition. The primary goal of therapy is symptom management. Therefore, the studies discussed in this review investigate the prevalence, types, and assessment of OPD and swallowing disorders in patients with CP as well as its impact on nutritional status and related health complications. Twelve articles deal with the challenges and management of dysphagia and swallowing disorders in CP [83–94]. Fourteen articles explore the nutritional status and gastroesophageal symptoms in CP [95–108] whereas the other fifteen papers examine the assessment techniques and rehabilitation strategies for the nutritional outcome in individuals with CP [109–123].

### Impact of Dysphagia on Nutrition and Swallowing Disorders in Cerebral Palsy: Challenges and Management

Dysphagia and swallowing difficulties in CP pose intricate challenges affecting health and quality of life, necessitating specific management approaches. In the first five studies, it is consistently found that dysphagia is very common among people with CP and is frequently linked to both reduced functioning and a lower quality of life. Different levels of dysphagia symptoms are seen depending on the seriousness and stage of swallowing difficulties, with more noticeable problems seen in individuals with severe motor restrictions. In an initial cross-sectional examination, 81.5% of 103 children with CP experienced dysphagia, with 31% displaying tracheal aspiration during videofluoroscopy, but none contracted aspiration pneumonia, highlighting the procedure's safety within this group [83]. This study revealed a high prevalence of OPD in children with CP, with 81.5% of participants exhibiting some degree of swallowing dysfunction. This high prevalence is clinically significant, as OPD is associated with adverse health outcomes such as growth delays due to inadequate nutritional intake and an increased risk of GER. An observational study comparing mastication in 8 children with spastic CP found notable delays in chewing and less synchronized tongue movements in comparison to typically developing children. This highlights the importance of using mastication observation, evaluation, and ultrasound assessments for personalized analysis [84]. In a study of 130 children with CP, Benfer et al. found that 67.7% had pharyngeal dysphagia, which was linked to lower gross motor function. Coughing was a frequent but not specific indicator, and parent reports were moderately accurate [85]. A cross-sectional study of 67 children with spastic CP found that over half of them had swallowing difficulties associated with severe speech problems and significant cognitive impairment, particularly when the throat was affected [86]. Yi and his team studied 117 adults with CP and found that dysphagia symptoms had a significant impact on their quality of life, with chewing problems and choking on food commonly reported; decreased functional status and increased age also affected quality of life scores [87]. Seven additional studies show important problems with eating and swallowing in individuals with CP, affecting their nutrition, growth, and overall health. Typical discoveries include a high occurrence of swallowing difficulties, ongoing dangers inhaling food or liquid, and connections between feeding struggles and motor function issues, indicating that customized, collaborative treatments are crucial for enhancing safety and nutritional results. In a randomized controlled trial, 27 children with CP and moderate eating issues didn't show significant improvements in eating efficiency or growth outcomes after receiving 10–20 weeks of oral sensorimotor therapy. Weight and skinfold measurements did not change despite the absence of catch-up growth, indicating that the severity of impairment is better reflected in eating efficiency than in response to treatment [88]. Another cross-sectional study involving 50 children with CP and gastrointestinal issues found that GERD was present in 66% and OPD in 82%, especially among those with significant motor difficulties. After 6 months of receiving nutritional rehabilitation and therapy, only 46% showed weight gain, suggesting minimal progress [89]. In a group of 53 children diagnosed with CP at 18–24 months and 36 months, the prevalence of OPD stayed consistent, with 30% demonstrating progress. The severity of OPD was strongly predicted by gross motor function linked with lower weight and BMI, underscoring the importance of screening focused on gross motor skills to inform intervention approaches [90]. A study following 179 children with CP found that the prevalence of OPD decreased from 79.7% at 18–24 months to 43.5% at 60 months. The progress was mainly seen in kids with minor motor difficulties, highlighting the chance for natural improvement in less severe instances and the need for targeted treatments in more severe cases [91]. Seo and colleagues found that in 17 adults with dyskinetic CP and cervical dystonia, videofluoroscopic assessments showed a high prevalence of difficulties with chewing, tongue movements, and laryngeal elevation. Challenges in the pharyngeal stage were associated with the severity of cervical dystonia rather than the Gross Motor Function Classification System (GMFCS) level, highlighting the need for dysphagia screening in adults with severe cervical dystonia to improve treatment strategies [92]. Mouilly and his team studied 65 children with CP (ages 2–17) and found that growth delays were linked to gross motor dysfunctions and feeding problems through anthropometric analysis. Around 65% of individuals suffered from incorrect mouth openings and other facial deformities, leading to poor nutritional levels [93]. In comparison research, eleven children with spastic CP showed significant difficulties with eating and swallowing, such as heightened activity in the suprahyoid muscles and irregular coordination between breathing and swallowing, when compared to a control group. These issues, especially inhalation after swallowing, were found to be associated with more severe dysphagia scores, emphasizing the importance of focused interventions combining respiratory and swallowing techniques [94]. The findings from this subsection underscore the pervasive impact of dysphagia on individuals with CP, revealing significant challenges across various aspects of nutrition, motor function, and quality of life. Studies highlight the high prevalence of swallowing difficulties, their correlation with gross motor impairments, and their implications for growth and health outcomes. While some progress is observed in less severe cases, targeted and interdisciplinary management strategies remain essential for addressing the multifaceted needs of this population.

### Health risks Associated with OPD and Gastroesophageal Symptoms in Individuals with Cerebral Palsy

Ensuring proper nutrition for people with CP is a significant difficulty, influenced by distinct gastroesophageal symptoms and obstacles that require focused evaluation and rehab efforts. In the initial five studies on nutritional status in children with CP, it was consistently observed that they experience significant challenges in terms of nutrition and growth. These difficulties are influenced by age, severity of motor functions, and changeable lifestyle factors. Managing nutritional health in this population is complex due to growth abnormalities, changes in body composition, and a high occurrence of feeding and gastrointestinal problems. Wang and his team [95] assessed the nutrition status of 377 children with CP between the ages of 2 and 18 in West China, revealing that 42.4% were stunted, 21.5% were underweight, and 18.5% were overweight or obese. Older children and those with severe motor impairments showed more prominent nutritional deficiencies, which were closely linked to GMFCS and Manual Ability Classification System (MACS) levels, indicating age and motor functions as important factors for personalized nutritional strategies [95]. Bell et al. [96] conducted a longitudinal study on 240 young children with CP in Australia, ages 18 months to 5 years, and found a connection between dietary intake, sedentary behavior, and growth results. Adjustable aspects such as eating habits and exercise were associated with better body composition and development, underscoring the opportunity for lifestyle changes to improve health, well-being, and engagement [96]. A different long-term research project tracked 161 children with CP between 18 and 60 months old, revealing that children in GMFCS levels IV and V exhibited less fat-free mass and a higher body fat percentage than children with milder impairments. Energy consumption and regular physical exercise in children with GMFCS level I had a positive effect on body composition, highlighting the importance of personalized diet and exercise routines [97]. Another paper compared behavioral interventions for feeding difficulties in children aged 1–12 with CP and autism. Behavioral methods, including escape extinction and differential reinforcement, successfully decreased food rejection and boosted consumption in both groups, emphasizing the significance of behavioral techniques in managing feeding difficulties in CP [98]. A cross-sectional study looked at GERD and dental erosion in 46 children with CP aged 3–13, finding higher rates of GERD-related dental erosion and reduced saliva in those with GERD. Logistic regression analysis found a link between GERD and dental erosion, highlighting the importance of managing GERD to prevent oral health problems in this high-risk group [99]. Dysphagia, GER, and other feeding issues are frequently seen in children with CP, affecting their nutritional health and overall well-being. The degree of feeding and swallowing problems is linked to the severity of motor deficits, requiring customized evaluation and treatment strategies to effectively manage these complex issues. During a prospective cohort study of 29 children with CP aged 2 to 10 years, 3,899 reflux episodes were observed through 24-h multiple intraluminal impedance-pH monitoring, with 70% being non-acidic. This indicates that numerous instances of reflux in individuals with chronic pancreatitis may go unnoticed with just pH monitoring, impacting the treatment of GER and potentially exacerbating respiratory and nutritional issues [100]. In another study, 99% of the 166 children with severe CP, who had an average age of 9 years, displayed symptoms of dysphagia. Surprisingly, 76% of these individuals faced some level of challenge with swallowing, with 15% requiring tube feeding due to severe difficulties. The level of dysphagia was discovered to be strongly correlated with motor challenges rather than parental reports of feeding problems. This suggests parents may not be aware of the problem and stresses the need for early dysphagia screening [101]. In the research by Schepers et al. [102], it was observed that among 77 children with CP (average age: 7 years), those positioned in higher levels of the Eating and Drinking Ability Classification System (EDACS) experienced significantly fewer dysphagia restrictions, with EDACS explaining 55% of the variability. These results indicate that including dysphagia limitations in the thorough assessment of swallowing can assist in determining suitable feeding approaches [102]. In a study that looked at different age groups, dysphagia was common in a large portion of 82.5% of the 40 children with CP, while GER was present in 40% and constipation in 60% of the subjects. Kids with dysphagia and digestive issues displayed unique eating patterns that impacted their growth and overall nutrition. People with GER drank more fluids, while people with constipation had a lower intake of fiber and fluids, indicating the need for specific dietary interventions for different gastrointestinal symptoms [103]. A longitudinal study that followed 23 children with CP for 30 months found that issues with feeding remained stable, with minor changes in the occurrence of coughing. Children with significant difficulties in mouth movements experienced more eating difficulties/problems than those with minor or no issues/difficulties, highlighting the pervasive and enduring nature of feeding problems in CP and underscoring the necessity for ongoing support for children with severe disabilities [103]. The last four studies emphasize the intricate connection between CP, dysphagia, and nutritional status, suggesting that as motor and eating impairments worsen, nutritional difficulties also escalate. Performance in eating and drinking, length of mealtime, and need for help are key factors that affect nutrition results in individuals with CP, regardless of age. In a cross-sectional study of 17 children with spastic CP, mealtime duration measures were reliably associated with feeding performance. Children with unilateral brain involvement had shorter mealtimes and higher feeding scores, indicating a connection between motor impairment severity and feeding efficiency [104]. In a study by Zhao and colleagues, high levels of undernutrition were discovered in a group of 1,151 children, especially among those with severe limitations in motor skills and eating habits. The likelihood of moderate to severe undernutrition significantly increased in children with GMFCS and EDACS levels IV and V [106]. Oliveira and colleagues [107] conducted a study on 56 adult CP patients hospitalized for a year, finding that more severe dysphagia and dietary restrictions resulted in worse nutritional outcomes, despite no significant weight change over time [107]. Finally, a study by McAllister et al. [108] involving 2,035 adults demonstrated a link between severe EDACS levels and lower body weight and body mass index. 86% of patients with EDACS level V required gastrostomies, highlighting the importance of regular dysphagia screening and nutritional support for this group [108]. Overall, a strong association between motor impairments and OPD severity was found, with delayed pharyngeal transit time and laryngeal elevation issues commonly reported. Furthermore, key findings highlight the significant challenges in nutritional health, growth, and feeding efficiency, all closely linked to the severity of motor impairments and gastrointestinal complications. The need for personalized approaches to nutrition, lifestyle modifications, and targeted behavioral interventions is evident. Moreover, the association between GER, dysphagia, and oral health emphasizes the importance of comprehensive evaluation and early screening to mitigate long-term health risks.

### Diagnostic Tools for evaluating Nutritional Outcome in Cerebral Palsy: Assessment Techniques and Rehabilitation Strategies

**Fig. 4:**
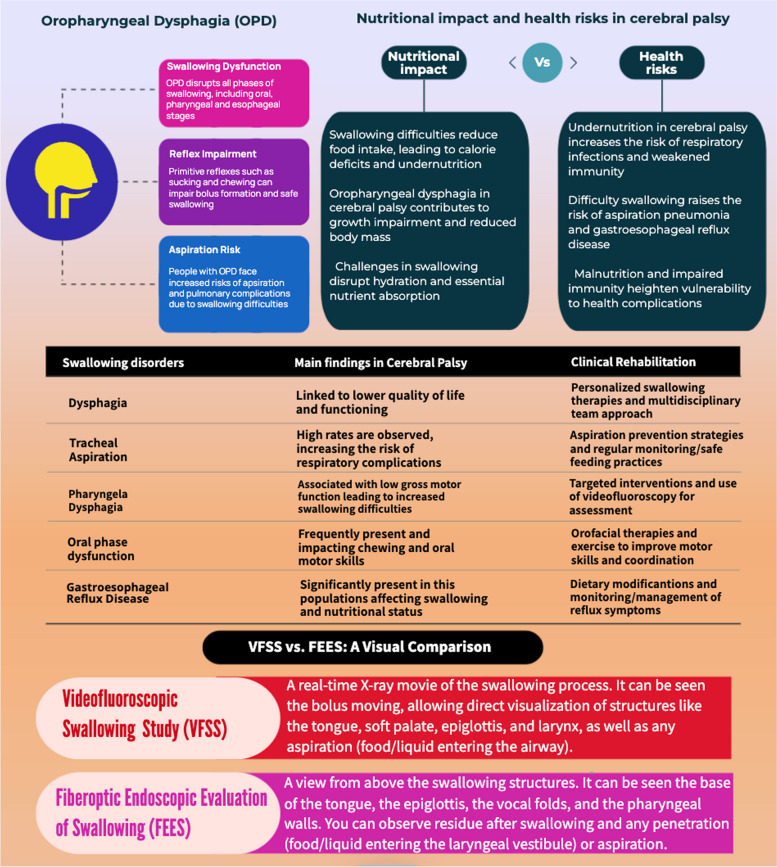
Key insights of results

Assessing nutritional improvements in individuals with CP requires a comprehensive approach combining certain evaluation methods with customized rehab plans to address their unique needs. The studies discussed in this paragraph emphasize the notable frequency of dysphagia and related nutritional difficulties in children with CP. Every research highlights the importance of complete evaluations and specialized interventions for this group of people. Santoro et al. [109] analyzed 40 children with CP and found that problems with oral motor skills cause issues with eating, malnourishment, and higher chances of inhaling food or liquid. The authors recommend a comprehensive evaluation from multiple disciplines, which involves neurological assessments, in order to create customized intervention plans [109]. Kantarcigil and colleagues [110] concentrated on assessing the dependability of asynchronous telehealth assessments for dysphagia in a sample of 19 children, aged between 6.9 and 17.5 years old. Findings revealed significant agreement between remote and in-person evaluations, demonstrating that telehealth is a viable choice for assessing dysphagia in areas with limited access to healthcare services [110]. Araujo and their team [111] examined the nutritional status of 187 children with CP. The study revealed that 10% of children were below the 10th percentile in weight when using CP-specific growth charts, showing that general pediatric norms frequently overestimate malnutrition. Furthermore, there were noteworthy connections discovered among dysphagia, constipation, and inadequate nutritional status [111]. Alacam et al. [112] conducted a study with 84 children, with 42 having CP and 42 being healthy controls, using the Nordic Orofacial Test-Screening. The results showed higher dysfunction scores in the CP group, especially in facial expression and swallowing, highlighting the importance of specialized orofacial therapies [112]. Costa and his group [113] evaluated 33 students at a special needs school, finding that every single one showed signs of oropharyngeal dysphagia, with 90.6% experiencing compromised swallowing safety and significant levels of malnutrition and dehydration. This research emphasizes the significance of utilizing multidisciplinary methods to effectively address the complex needs of this group [113]. A prospective study with 151 children with CP (average age 6.11 years) investigated how different dysphagia classification systems are related to functional profiles. Findings showed that 15.2% of participants had prominent swallowing difficulties, with clear connections between the EDACS, communication and gross motor skills, emphasizing the need for a comprehensive evaluation [114]. A study validating the EDACS system for evaluating eating and drinking skills in CP patients involved 151 participants. The research discovered a noteworthy 78% agreement among speech and language therapists, showcasing the system dependability and possibility for broad clinical use [115]. In a cross-sectional study the feeding and swallowing difficulties of 89 children with CP, aged averagely 6 years old, were evaluated. A screening tool with four items based on parent responses had a high sensitivity (81%) and specificity (79%) in identifying feeding problems, allowing for prompt interventions for affected children [116]. In a study comparing the Exeter Dysphagia Assessment Technique (EDAT) with comprehensive clinical assessments, 18 children with CP were evaluated. The EDAT showed substantial consistency (minimum 78%) in different feeding abilities, providing a non-intrusive and easy-to-use option for assessing dysphagia in kids [117]. A prospective study evaluated how well EDACS can identify the risk of aspiration in 131 children with CP, who had a median age of 4.4 years. The EDACS effectively detected the risk of aspiration, showing a sensitivity of 78% and specificity of 92%. This research backs the suggestion to utilize both EDACS and the Pediatric Eating Assessment Tool for the best clinical decision-making on swallowing studies [118]. Su et al. [119] studied 16 severely affected children with CP at levels IV and V, comparing the Mann Assessment of Swallowing Ability (MASA) with videofluoroscopic swallowing studies. Findings demonstrated that although MASA did not distinguish between aspirators and nonaspirators, it was able to predict oral dysfunction, suggesting its restricted usefulness in evaluating aspiration [119]. An experiment with random allocation tested the effects of oral sensorimotor stimulation on 71 children between 12 and 48 months old who have spastic quadriplegia. The intervention resulted in notable enhancements in oral motor function and physical growth in comparison to traditional therapy, underscoring its effectiveness in improving feeding abilities [120]. Sousa and colleagues [121] conducted research on 28 patients with spastic quadriplegic CP who were under the age of 13. The research discovered that 75% used different feeding methods, with 57% classified as eutrophic. Thus, a significant relationship was found between dietary intake and nutritional status, underscoring the necessity of personalized nutritional advice [121]. Yilmzaz and his team [122] evaluated functional feeding abilities in a group ranging from 4 to 25 years old using the Modified Functional Feeding Assessment Scale. While parents did not notice major feeding problems, the findings showed significant struggles in different feeding abilities, emphasizing a difference between what parents thought and the actual issues [122]. A last randomized controlled trial assessed the viability of the babiEAT intervention in 14 infants with CP and oropharyngeal dysphagia. Both babiEAT and standard care were deemed satisfactory by parents, but they expressed greater satisfaction and perceived effectiveness with babiEAT. Additionally, babiEAT improved fluid intake efficiency and food texture acceptance without raising the risk of aspiration [123]. Overall, the evidence emphasizes the critical role of comprehensive diagnostic approaches and individualized treatment plans in addressing the high prevalence of dysphagia and nutritional difficulties among children with CP. Emerging tools, such as telehealth for dysphagia assessments and standardized instruments like EDACS and EDAT, show promise in improving diagnostic accuracy and clinical decision-making. The importance of tailored approaches, including oral sensorimotor stimulation and individualized dietary planning, was also evident, demonstrating potential for improving both nutritional outcomes and quality of life in this population. Key insights of the results section are presented in Fig. 4.

## Discussion

**Fig. 5:**
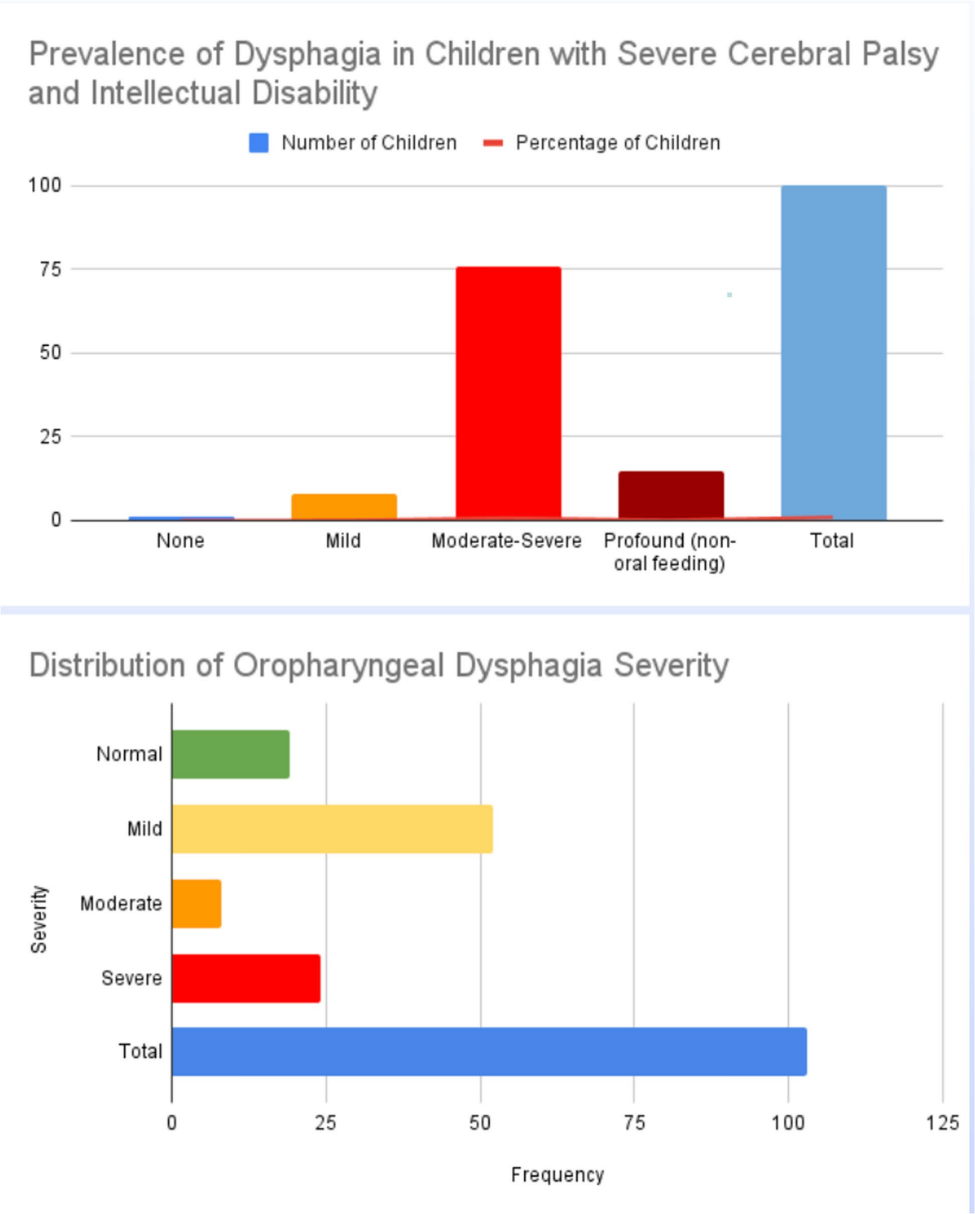
Prevalence of OPD and Dysphagia in Children with CP

This systematic review examined the prevalence, types, and assessment of OPD and swallowing disorders in patients with CP as well as its impact on nutritional status and related health complications. The results concerning dysphagia and swallowing issues in people with CP highlight the intricate and diverse nature of these difficulties, mirroring trends seen in previous research [126–128]. Our review consistently found high prevalence rates of dysphagia in CP, with some studies reporting rates exceeding 80% [83, 89, 101, 103] as showed in Fig. 5. This is consistent with previous literature using videofluoroscopy as the diagnostic tool, which has also reported high prevalence. However, it is important to note that studies using other methods, such as FEES may report different prevalence rates. While VFSS allows for visualization of all phases of swallowing and is considered the gold standard, FEES offers advantages in portability and the ability to assess sensory function, but is limited in visualizing the oral phase. The connection between dysphagia and the extent of motor disabilities, as demonstrated in various studies within our review [85, 90, 101], is also consistent with prior work [129, 130]. This relationship underscores the impact of motor control on the complex coordination required for safe and efficient swallowing. Studies have shown that problems with swallowing can hinder both quality of life and overall health [78, 87, 131]. Specifically, difficulties with chewing, as reported in several of the included studies [84, 87, 92], can lead to reduced food intake and subsequent nutritional deficiencies. Similarly, the frequent occurrence of coughing and aspiration [83, 85, 94] highlights the risk of respiratory complications, which can further impact health and well-being. This is consistent with existing literature highlighting the link between aspiration and increased risk of pneumonia in individuals with neurological impairments [132, 133]. Several studies have pointed out that traditional interventions may not be sufficient in improving eating efficiency or growth outcomes in individuals with CP [88, 89], indicating that standard therapies may not tackle the root complexities of dysphagia. For instance, the study by Gisel et al. [88] found no significant improvements in eating efficiency or growth outcomes after oral sensorimotor therapy, which aligns with findings from other research suggesting that more intensive or combined interventions may be necessary [134, 135]. This highlights the need for further research to explore the effectiveness of different therapeutic approaches, including combinations of oral motor exercises, postural adjustments, and dietary modifications. The results underscore the importance of thorough evaluations, which should include clinical observations and instrumental assessments like videofluoroscopy and ultrasound, to create tailored treatment strategies that address the specific challenges associated with chewing and swallowing [136]. Moreover, the high occurrence of gastroesophageal symptoms in children with CP and their relation to nutritional health highlight an important aspect that needs more attention in clinical practice. The ongoing recognition of GERD and OPD as major concerns indicates a need for holistic approaches to address both swallowing disorders and nutritional deficits [137]. This is particularly relevant considering the high prevalence of GERD (66% in one study) [89] and its potential to exacerbate swallowing difficulties. The prevalence of undernutrition and obesity in this group is a result of how motor functions, feeding habits, and health outcomes are interconnected, highlighting the significance of personalized lifestyle changes and dietary interventions. In summary, these results add to the current understanding by emphasizing the ongoing issues related to dysphagia in CP and promote a change towards inclusive, collaborative treatment strategies. New rehabilitation strategies need to focus on constantly assessing and adjusting to the specific requirements of individuals with CP, with the goal of enhancing their well-being and quality of life. It is important to note that, even with differences in environmental factors, dietary practices, and access to healthcare, research consistently demonstrates that OPD impacts a similar percentage of children with CP in various nations. This consistency emphasizes the core characteristics of swallowing challenges in CP and indicates that OPD is strongly linked to the neurological and physiological obstacles that come with the condition rather than outside influences. The widespread occurrence of OPD showcases the importance of standardized evaluation and treatment plans that can be used globally, guaranteeing that all children with CP receive the required assistance to address their feeding concerns securely.

### A Multidisciplinary Approach to Dysphagia in CP: from Diagnosis to Personalized Intervention Strategies

Beyond the established principles of early detection and multidisciplinary care, this review underscores the critical need for more structured and individualized approaches to dysphagia management in CP. A key advancement would be the development and implementation of standardized diagnostic protocols for OPD. Current practice often varies significantly, hindering consistent diagnosis and treatment planning. Standardized guidelines, incorporating clinical observations, instrumental assessments like VFSS and FEES, and validated questionnaires, while accounting for individual motor, cognitive, and communicative abilities, would greatly enhance diagnostic accuracy and facilitate inter-clinician communication [138, 139]. This, in turn, allows for the creation of truly tailored interventions. For example, specific postural adjustments like side-lying or chin tuck, depending on the nature of the swallowing impairment, can significantly improve swallowing safety. Similarly, adaptive utensils, such as angled spoons or non-slip grips, can empower individuals with limited motor control to participate more actively in mealtimes. Texture-modified diets, ranging from pureed to mechanically altered, should be carefully prescribed by a dietitian in collaboration with the speech-language pathologist, ensuring both nutritional adequacy and swallowing safety. Furthermore, fostering robust multidisciplinary collaboration is paramount [140]. An ideal care model would involve regular joint meetings between neurologists/developmental pediatricians (managing the underlying neurological condition), speech-language pathologists (addressing swallowing mechanics and oral motor skills), dietitians (optimizing nutritional intake and managing dietary modifications), and, crucially, the caregivers. These meetings would facilitate shared goal setting, consistent implementation of strategies across settings, and ongoing monitoring of progress. For instance, if a child is identified as having difficulty with the pharyngeal phase of swallowing during VFSS, the speech-language pathologist can implement specific exercises and strategies, while the dietitian adjusts the diet to appropriate consistencies, and the caregivers are trained on safe feeding techniques and positioning to reinforce these strategies at home. Finally, future research should explore the potential of emerging technologies to address challenges, particularly in low-resource settings. Portable videofluoroscopy offers the possibility of bringing instrumental assessments to remote communities, while artificial intelligence (AI)-driven diagnostic tools could enhance the efficiency and accessibility of dysphagia screening, potentially identifying individuals at risk earlier and allowing for more timely intervention. By analyzing patient data and clinical inputs, AI algorithms could quickly and accurately detect early signs of swallowing dysfunction, facilitating faster and more reliable diagnoses. This would be particularly valuable in areas with fewer specialized clinicians, allowing for more widespread and efficient identification of at-risk individuals. Furthermore, the integration of these technologies could drive cost-effectiveness by reducing the need for frequent in-person specialist consultations and enabling remote monitoring. These technological advancements, coupled with standardized protocols and robust multidisciplinary collaboration, hold significant promise for transforming the landscape of dysphagia management in individuals with CP.

### Strengths and Limitations

This thorough review provides a strong summary of current research on OPD in children with CP, offering important perspectives for clinical practice and guiding future research. The review's rigorous search strategy, which utilized several reputable databases without time limitations, is a key strength as it enables a comprehensive analysis of the changing understanding of OPD and swallowing disorder in this group. The utilization of the PRISMA guidelines improves transparency and methodological rigor by providing detailed information on the study selection process. Having several evaluating studies independently, along with calculating inter-rater reliability using the kappa statistics, enhances the trustworthiness of the data extraction process and minimizes potential bias. Moreover, incorporating both quantitative and qualitative research enhances the results of the analysis by covering a diverse array of experiences concerning swallowing disorders and its effects on nutritional status and health issues. Analyzing a particular group, which includes children and adults with CP, guarantees that the findings are relevant to this vulnerable population. This underscores important care strategies for healthcare professionals and caregivers. Nevertheless, some constraints are present in this systematic review. The exclusion of non-English studies may have led to the omission of significant research, which could limit the generalizability of the findings. This limitation may lead to an inadequate representation of the worldwide research environment for various populations and contexts. Additionally, omitting non-English studies might unintentionally create a geographical or cultural bias, thereby limiting the relevance of the findings to a particular group of countries or populations. The evaluation of bias through tools such as ROBINS-I and RoB 2, though necessary, raised issues pertaining to study design, particularly concerning the selection and reporting biases in certain studies that were included. Furthermore, the reliance on observational studies, which lack comparison groups, may reduce the ability to draw definitive conclusions about the causal relationships between swallowing disorders and nutritional outcomes. To address these gaps, future research should aim to include studies in a broader range of languages and prioritize high-quality randomized controlled trials or cohort studies with comparison groups to strengthen the evidence base and improve clinical recommendations.

## Conclusions

This systematic review unequivocally confirms the widespread prevalence and significant clinical ramifications of OPD and related swallowing disorders in individuals with CP. The consistently high prevalence of dysphagia observed across studies, frequently exceeding 80%, underscores the critical need for improved clinical practice. These swallowing difficulties are not isolated issues; they profoundly impact nutritional status, growth, overall health, and quality of life, often compounding risks such as GER and respiratory complications. Therefore, this review strongly advocates for several key actions. First, clinicians should prioritize the implementation of standardized diagnostic protocols for OPD in CP, incorporating both clinical observation and instrumental assessments like VFSS and, where appropriate, FEES, to ensure accurate diagnosis and facilitate effective treatment planning. Second, truly integrated multidisciplinary care models are essential. This necessitates close collaboration among neurologists/developmental pediatricians, speech-language pathologists, dietitians, physical therapists, and, crucially, caregivers, to ensure consistent and holistic management. Finally, this review highlights the transformative potential of emerging technologies. Portable VFSS can improve access to instrumental assessments, especially in resource-limited settings, while AI-driven diagnostic tools offer the promise of more efficient and objective screening, enabling earlier identification and intervention. By implementing these practical steps (standardizing diagnosis, fostering multidisciplinary collaboration, and embracing technological advancements) clinicians and researchers can profoundly improve the lives of individuals with CP and dysphagia, mitigating health risks and enhancing overall well-being.

## Data Availability

The data that support the findings of this study are not openly available due to reasons of sensitivity and are available from the corresponding author upon reasonable request.

## Abbreviations

CP: Cerebral Palsy
EDACS: Eating and Drinking Ability Classification System
EDAT: Exeter Dysphagia Assessment Technique
FEES: Fiberoptic Endoscopic Evaluation of Swallowing
GER: Gastroesophageal Reflux
GERD: Gastroesophageal Reflux Disease
GMFCS: Gross Motor Function Classification System
MACS: Manual Ability Classification System
MASA: Mann Assessment of Swallowing Ability
OPD: Oropharyngeal Dysphagia
VFSS: Videofluoroscopic Swallowing Studies
